# Biological and Race Strategy Characteristics Investigated in Elite Endurance Track Running: A Systematic Review

**DOI:** 10.1007/s40279-026-02475-8

**Published:** 2026-07-09

**Authors:** Tarryn M. Elvish, Danielle Trowell, Jason Bonacci, Kirstie J. Turner, Peter Kremer, Emma L. Millett, Craig Pickering, Natalie Saunders

**Affiliations:** 1 Centre for Sport Research, Deakin University, Waurn Ponds, VIC 3215 Australia; 2 Australian Athletics, Melbourne, VIC 3206 Australia; 3 NSW Institute of Sport, Sydney, NSW 2127 Australia

## Abstract

**Background:**

Understanding biological and race strategy characteristics of endurance track runners competing at benchmark events provides high-performance athletics programmes and coaches with valuable insight into what is required to succeed.

**Objectives:**

The aim of this systematic review was to identify biological and race strategy characteristics investigated in elite able-bodied endurance track runners (Aim 1) and, where examined, highlight whether these characteristics could differentiate performance success within this elite cohort (Aim 2).

**Methods:**

For this systematic review, searches across EBSCOhost (Academic Search Complete, CINAHL Complete, MEDLINE Complete and SPORTDiscus), Scopus, PubMed and Web of Science were completed until May 2023. Searches also involved World Athletics Research Centre, New Studies in Athletics, handsearching and scanning of reference lists of included studies. Eligible studies were required to be published in English and have investigated performance-related biological or race strategy characteristics among senior able-bodied endurance track runners (800–10,000 m) capable of competing at benchmark events. Risk of bias of included studies was evaluated based on Sarmento et al.’s 16-item checklist. The review separated middle-distance runners (MDR) and long-distance runners (LDR), to account for the differing performance demands of these event groups. Studies were considered to address Aim 2 if they assessed for statistical associations between athlete characteristics and overall performance (e.g. race time) or compared characteristics between athletes with different competitive results (e.g. medallists and non-medallists).

**Results:**

Thirty-nine articles were included in this review, with 23 and 21 of these articles relevant to MDR and LDR, respectively. The most commonly reported characteristics were related to anthropometry (7 studies for MDR and LDR), biomechanics (7 studies for LDR), pacing (9 and 10 studies for MDR and LDR, respectively), qualification pattern (6 studies for MDR) and benchmark performance relative to athlete history (9 and 7 studies for MDR and LDR, respectively). However, the diversity of biological characteristics investigated and the generally small sample sizes makes it difficult to define an optimal biological profile for elite endurance runners. In contrast, race strategy characteristics investigated often included larger sample sizes providing a better understanding, especially in relation to pacing and for middle-distance events qualification patterns. Further, studies investigating whether characteristics differentiate performance among elite endurance track runners indicate that the most successful runners consistently demonstrate superior finishing ability. This is reflected in faster speeds during at least one 100-m segment of the final 400 m of the race and the ability to complete the final segments of the race at a pace close to, or faster than, their season best or 32-month best performance. In long distance events, better performance was associated with faster personal best times across the previous 32 months, indicating that having a high-ranking recent best time may be a prerequisite for succeeding at benchmark events. Faster 10,000-m runners had lower body mass and smaller arm and calf circumferences, suggesting a potential advantage of having lower non-functional mass. In middle distance events, success was associated with achieving superior qualification positions in the heats and semi-final, highlighting the importance of tactical positioning and efficient progression through qualification rounds. Faster male 800-m runners had a technique characterised by longer contact times and more compliant spring mechanics which may allow athletes to maintain or elevate speed during the sprint finish.

**Conclusion:**

Biological and race strategy characteristics that profile elite endurance track runners and that, in several studies, showed association with performance success were discussed. These findings may assist high-performance athletics programmes and coaches in informing athlete development and investment. However, further research is required to establish a more holistic understanding of the biological profile of elite endurance track athletes and to determine which biological and race strategy characteristics may differentiate performance reliably among these elite runners.

**Supplementary Information:**

The online version contains supplementary material available at https://doi.org/10.1007/s40279-026-02475-8.

## Key Points

**Table:** 

Optimal biological characteristics of elite endurance track runners remain unclear. Further research is needed to better understand the biological profile of elite endurance track runners.
Superior capacity to maintain race speed in the latter stages of the race (faster 100-m segments in the final 400 m) consistently differentiated top performers.
In middle-distance running, success at benchmark events was associated with achieving superior qualification positions, highlighting the importance of tactical positioning and efficient progression through qualification rounds.
In long-distance running, success was associated with faster recent personal best times, suggesting that high-ranking recent times may be essential for succeeding at benchmark events.
Further research is required to determine which biological and race strategy characteristics may differentiate performance reliably among elite endurance track runners.

## Introduction

High-performance sporting organisations are responsible for identifying and developing future elite athletes with the goal of achieving international sporting success. Understanding the profile of elite athletes and characteristics associated with success is an essential first step. Previous systematic reviews have investigated performance-related characteristics specific to many different sports [1–10]. Recently, a systematic review investigated characteristics associated with success in elite speed and power-based track and field events [10]. Building on this research, the present systematic review investigates characteristics associated with success in elite able-bodied endurance-based track running events. Specifically, this review focuses on middle- (800-m and 1500-m) and long-distance (3000-m steeplechase, 5000-m and 10,000-m) track running events.

Previous studies have identified biological characteristics capable of differentiating performance among endurance track runners. Greater physiological capacity [11–19], optimal biomechanics [20] and anthropometric profile [21–23] and greater power [11, 24] have all been associated with better endurance running performance among trained to near-elite endurance track runners. For example, greater velocity at maximal oxygen uptake ($\dot{V}$O₂max) [13, 14, 17, 18] and greater speed at lactate threshold [13, 18, 19] have been associated with better endurance running performance. Further, lower skinfold thickness [23], greater lower leg mass to upper leg mass ratio and greater total body lean mass [21] have been associated with better middle-distance running performance. Faster sprint times [11, 24] and greater maximal sprint speed [25, 26] have also been associated with better endurance running performance, mainly middle-distance running performance [24–26]. Race strategy characteristics are also important for success in endurance track running events [27–30], with athletes required to manage pace fluctuation, respond to surges and strategically distribute their energy throughout the race [27]. Common pacing profiles seem to be associated with specific endurance track running events [27]. Tactical behaviours including running close to the inside of the lane and matching variations in pace with race leaders have been identified as important for performance success [31]. Clearly, biological and race strategy characteristics play important roles in endurance track running performance.

Despite substantial research investigating biological and race strategy characteristics associated with performance in endurance track running, it remains unclear what is required to compete and succeed at benchmark events, specifically the Olympics and World Championships. For example, the above studies identifying biological characteristics associated with performance do not include athletes capable of competing at these benchmark events [11–26], despite some studies defining their athletes as elite [11, 14, 15, 22, 23, 25, 26]. Sport science research often classifies participants as ‘elite’ with considerable inconsistency in who that term includes (from regional competitors to Olympic medallists) and in the criteria used (competition level, years of experience, weekly training volume) [32]. This distinction is important to specifically understand what is required to compete and succeed at benchmark events. For example, higher $\dot{V}$O₂max has consistently been associated with better performance among trained endurance runners [33–36], whereas several studies report no association among higher performing endurance runners [11, 15, 22, 37, 38]. This does not imply that $\dot{V}$O₂max is not important for elite endurance runners, but does suggest that elite runners likely reach a minimum $\dot{V}$O₂max threshold at which point improvements above this threshold no longer differentiate performance. Further, race strategies used at benchmark events often differ from other competitions because of the emphasis on race position over time and the absence of pacemakers [29, 30, 39]. Given the specific constraints of benchmark event performance, there is a need to specifically understand athletes capable of competing at this performance standard [40].

To understand what is required to compete and succeed at benchmark events in endurance track running, it is necessary to review existing evidence investigating biological and race strategy characteristics within this elite cohort. A greater understanding of these characteristics can support more targeted development and inform strategic investment decisions within high-performance programmes and coaching practice. Therefore, the aim of this study was to identify biological and race strategy characteristics investigated in elite able-bodied endurance track runners (Aim 1) and, where examined, highlight whether these characteristics could differentiate performance success within this elite cohort (Aim 2).

## Methods

This review was conducted in accordance with the Preferred Reporting Items for Systematic Reviews and Meta-analyses (PRISMA) guidelines [41].

### Eligibility Criteria

Studies were eligible for inclusion in this review if they met the following modified PICo approach [42]:

1. *Population*: Elite senior able-bodied track endurance runners (800–10,000 m). Athletes were classified as elite if, for biological studies, their mean performance times met the minimum qualification standard for benchmark events, specifically the Olympics and World Championships between 2016 and 2020 (Table 1), or, for biological and race strategy studies, if they placed within the top 16 of a benchmark event final including Olympics and World Championships since 1999.
2. *Phenomena of interest*: performance-related biological or race strategy characteristics.
3. *Context*: characteristics investigated or that differentiate performance.

**Table 1:** Minimum qualification standards for benchmark events, specifically the Olympics and World Championships between 2016 and 2020, for eligibility in this review

Event	Men (min:s)	Women (min:s)
800-m	1:46.00	2:01.50
1500-m	3:36.20	4:07.50
3000-m steeplechase	8:32.00	9:45.00
5000-m	13:25.00	15:24.00
10,000-m	28:00.00	32:15.00

Studies were required to be published in English and in an academic journal or by World Athletics Research Centre or New Studies in Athletics. Studies were excluded from this review if (1) only abstracts were provided; (2) they primarily discussed genetics or age; (3) participants were injured or returning from injury; (4) they were based on assessing the quality (validity or reliability) or comparing the quality of measures; (5) they focused on mathematical or theoretical modelling of performance; (6) they did not include participant performance times, highest competition standard or event specialisation and the author did not provide additional information; (7) they included some eligible participants, but the sample mean did not meet the criteria and no separate data were provided for eligible participants despite requests for additional information; (8) biological characteristics were not relevant to performance (e.g. medical assessment).

Studies investigating genetics associations with performance were excluded in line with the Australian Institute of Sport position statement, which states that genetic testing to predict performance is unscientific and unethical [43]. Age and training history were considered social characteristics. Studies based on mathematical or theoretical modelling were excluded as the purpose of this review was to investigate characteristics possessed by elite athletes, not to predict or estimate these characteristics based on performance times.

### Information Sources

EBSCOhost was used to search the following electronic databases: Academic Search Complete, CINAHL Complete, MEDLINE Complete and SPORTDiscus with Full Text. Additionally, Scopus, PubMed and Web of Science were also individually searched. Further grey literature was investigated through World Athletics Research Centre (https://www.worldathletics.org/about-iaaf/documents/research-centre) and New Studies in Athletics (https://worldathletics.org/nsa/popular/1/90) websites as these contained relevant reports providing information on technical aspects of athletics often not published in academic journals. All studies listed until May 2023 were considered for inclusion. All reference lists of included studies were reviewed for additional articles.

### Search Strategy

The search strategy was developed by the primary supervisor (NS) with guidance from a research librarian. A trial-and-error method was used to identify the most appropriate search terms. Key words were searched in the following areas: performance standard (elite or Olympic or champion or international), event specification (runn* or steeplechase) and with context (performance or differ* or compar* or characteristics or predict*). Due to nuances between different databases, the specific search strategies used were modified to suit each database. Terms ‘analysis’, ‘runn’, ‘800’, ‘1500’, ‘steeplechase’, ‘5000’, ‘10,000’, ‘middle-distance’, ‘long-distance’, ‘middle distance’, ‘long distance’, ‘performance’, ‘biomechanic’, ‘physiolog’, ‘anthropometr’, ‘speed’ and ‘predict’ were searched in New Studies in Athletics. All studies published in World Athletics Research Centre and by an author with research in this area were considered.

### Selection Process

One reviewer (TE) consolidated articles by removing duplicates and screening titles/abstracts/full text of all retrieved articles for eligibility. Duplicate articles were initially automatically removed by Mendeley, followed by a secondary check using the ‘check for duplicates tool’ with the reviewer TE confirming removal of true duplicates. Titles of studies from all databases were screened for eligibility. Potentially relevant studies were then assessed based on their abstracts, followed by full-text screening for final inclusion or exclusion. For studies published after 2000, authors were contacted if eligibility was unclear due to limited participant information or if the study included some eligible participants but did not report individual results. Studies in which eligibility was difficult to determine were discussed between reviewers (TE and NS) until consensus was reached. The reference list of included articles, as well as New Studies in Athletics and World Athletics Research Centre were searched for additional studies, and if relevant, these were screened by their abstract then full text. Two articles [44, 45] from World Athletics Research Centre were not included despite being eligible as they assessed the same athletes competing at the same event and analysed most of the same characteristics as the study already included in Hanley et al.’s study [46]. In cases of replication, the article assessing the greatest number of eligible athletes was retained.

### Data Collection Process and Data Items

One reviewer (TE) extracted data from each study, including type of study, specific biological or race strategy characteristics investigated and the number of athletes or athlete performances (race strategy studies only) for each event assessed. As reporting every individual characteristic was not feasible, specific characteristics (e.g. knee joint angle at contact) were grouped into broader categories (e.g. joint angles). The number of timepoints and the period over which each characteristic was measured were considered irrelevant to the purpose of this review and were therefore not included. For studies that also evaluated whether biological or race strategy characteristics could differentiate performance among elite endurance track runners (Aim 2), additional data were collated including author name, athletes’ performance time or relevant competition analysed, testing procedure used and overview of main outcomes. Additionally, the strength or magnitude of any statistically significant difference or association were included.

### Synthesis Methods

Studies were synthesised by event specialisation, middle distance (800-m and 1500-m) and long distance (3000-m steeple chase, 5000-m and 10,000-m). This classification aligns with Sandford and Stellingwerff’s study [47], which highlights distinct performance demands between events lasting 1.5–5 min compared with those of longer duration. Attempts to remove repeat athlete measures were only feasible for race strategy studies in which the same characteristics were investigated for the same group of athletes (e.g. medallists or finalists) at the same competition (e.g. World Championships 2017). When duplicate athlete entries were detected, they were counted once and removed from the total sample reporting that characteristic. In other cases, repeat measures could not be identified because athlete names were not provided. Consequently, some results may include the same athletes, but in different competitions or at different points in their career.

Studies were considered to investigate characteristics (Aim 1) if they reported performance-related biological or race strategy characteristics among eligible elite endurance track runners. Characteristics investigated in each study were grouped according to their theme under biological characteristics including morphology, anthropometry, physiology, and biomechanics, or race strategies including pacing, tactics, qualification pattern and benchmark performance relative to athlete performance history.

Studies were considered to evaluate whether characteristics could differentiate performance (Aim 2) if the study assessed for statistical associations between characteristics and overall performance or compared characteristics between athletes with different competitive results (e.g. medallists and non-medallists). Overall performance was required to reflect the athlete’s overall performance capacity or outcome including finishing position, finishing time, race speed, personal best (PB), or season best (SB) time. Race speed at a particular point in time was not included as it was not considered to reflect an athlete’s overall performance.

### Research Quality Assessment

A 16-item checklist, previously utilised in other sport science systematic reviews [2, 10, 48, 49], was selected as it best aligned with the competition-, field- and laboratory-based studies included in this review. Other commonly used research quality assessment methods did not allow for the same comparison between different study types. Therefore Sarmento et al.’s 16-item checklist [49] developed based on Law et al.’s original critical review form [50, 51] was used to assess the research quality. Research quality was defined as the extent to which a study minimised bias and was applicable to the review question [50, 51]. This checklist includes study purpose (1), adequate background literature (2), appropriate design (3), sample description (4), sample size justification (5), informed consent (6), measurement outcome reliability (7), measurement outcome validity (8), method description (9), reported results in terms of statistical significance (10), appropriate analysis methods (11), practical importance (12), reported drop-outs (13), appropriate conclusions (14), practical applications (15) and acknowledgement of study limitations (16). Item 13 (dropouts) was excluded from this review as only one study involved a follow-up procedure. The 15 remaining items were scored as 0 = no, 0.5 = partially addressed and 1 = yes, with a ‘not applicable’ option available for items 3, 6 and 10. Item 3 (appropriate design) was not applicable if no explicit aim or research question was stated for item 1. Item 6 (informed consent) was not applicable where studies used publicly available data, data owned and approved by World Athletics, or reports prepared for World Athletics. Item 10 (statistical significance) was not applicable in case studies assessing an athlete at a single timepoint (e.g. once during a testing session or once during an event), with no repeated observations available for comparison. All answers were summed, and the final score was divided by the maximum that the study could reach and expressed as a percentage to determine the study’s quality score. Studies scoring ≤ 50% were classified as low quality, studies scoring 51–75% were classified as good quality and studies scoring > 75% were classified as excellent quality [49]. Eligible studies were assessed by two members of the research team (TE and NS). Following independent scoring, differences were discussed until consensus was reached.

## Results and Discussion

### Study Selection

**Fig. 1:**
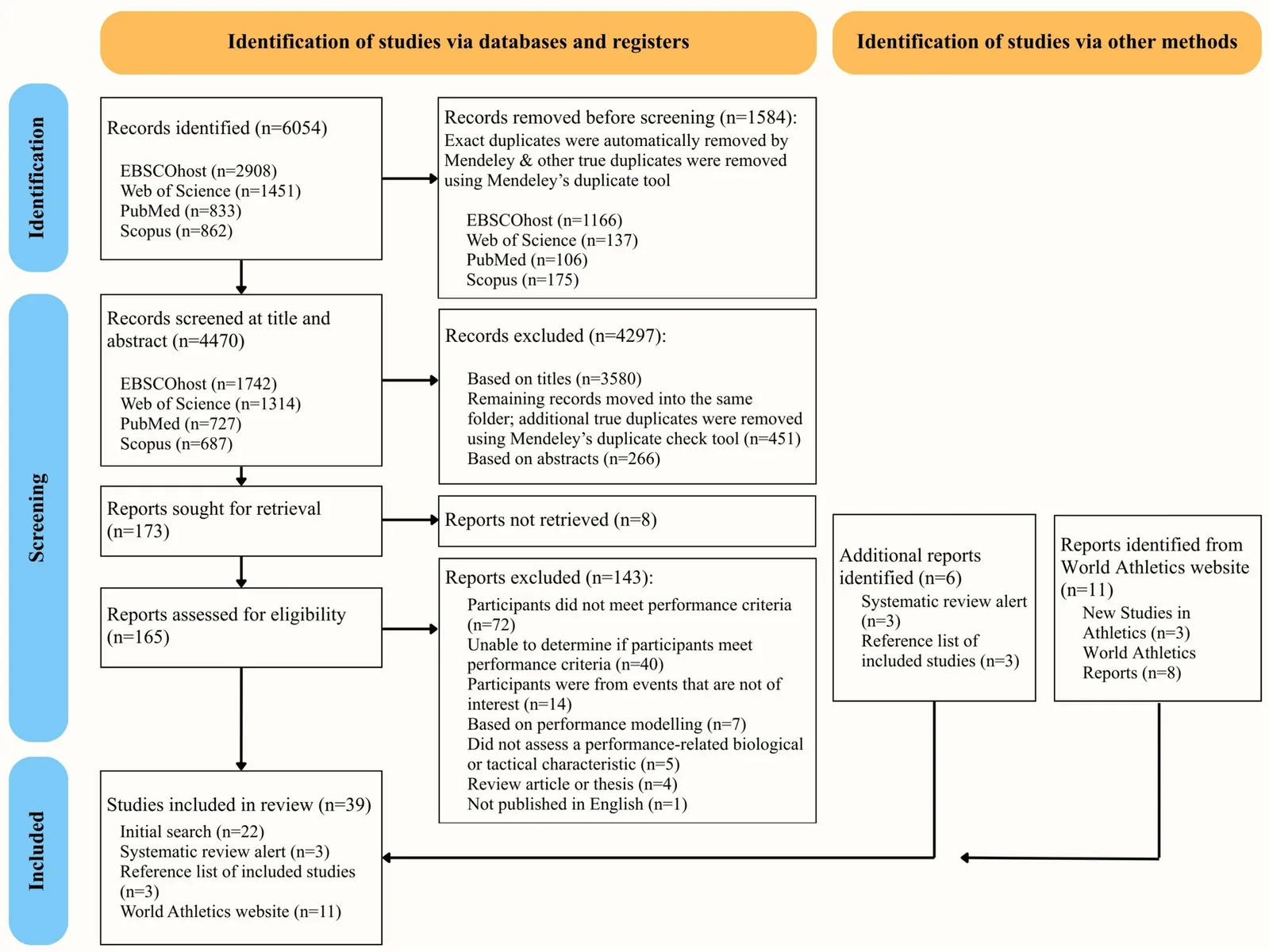
Preferred Reporting Items for Systematic Reviews and Meta-analyses (PRISMA) flowchart of the study selection process

Of the 6054 records identified through database searching, 22 were eligible. A further 17 eligible records were identified through World Athletics publications (*n* = 11), systematic review alerts (*n* = 3) and backward citation searching (*n* = 3) (Fig. 1). In total, 39 records were included in this review, with 23 and 21 of these articles relevant to middle-distance runners (MDR) and long-distance runners (LDR), respectively.

### Study Quality

Research quality of included studies was assessed using Sarmento et al.’s checklist [49]. The mean research quality score for the 39 studies was 67%. Seventeen studies were classified as excellent quality, while 11 articles were good quality and 11 articles were low quality. Recurrent quality issues included lack of justification of the study’s sample size, absence of addressing informed consent and failure to appropriately report the limitations of the study. A summary of each study’s quality score on all items of Sarmento et al.’s checklist [49] is provided in Supplementary Table 1 (see electronic supplementary material [ESM]).

### Middle-Distance Running

#### Results for Middle-Distance Running

**Fig. 2:**
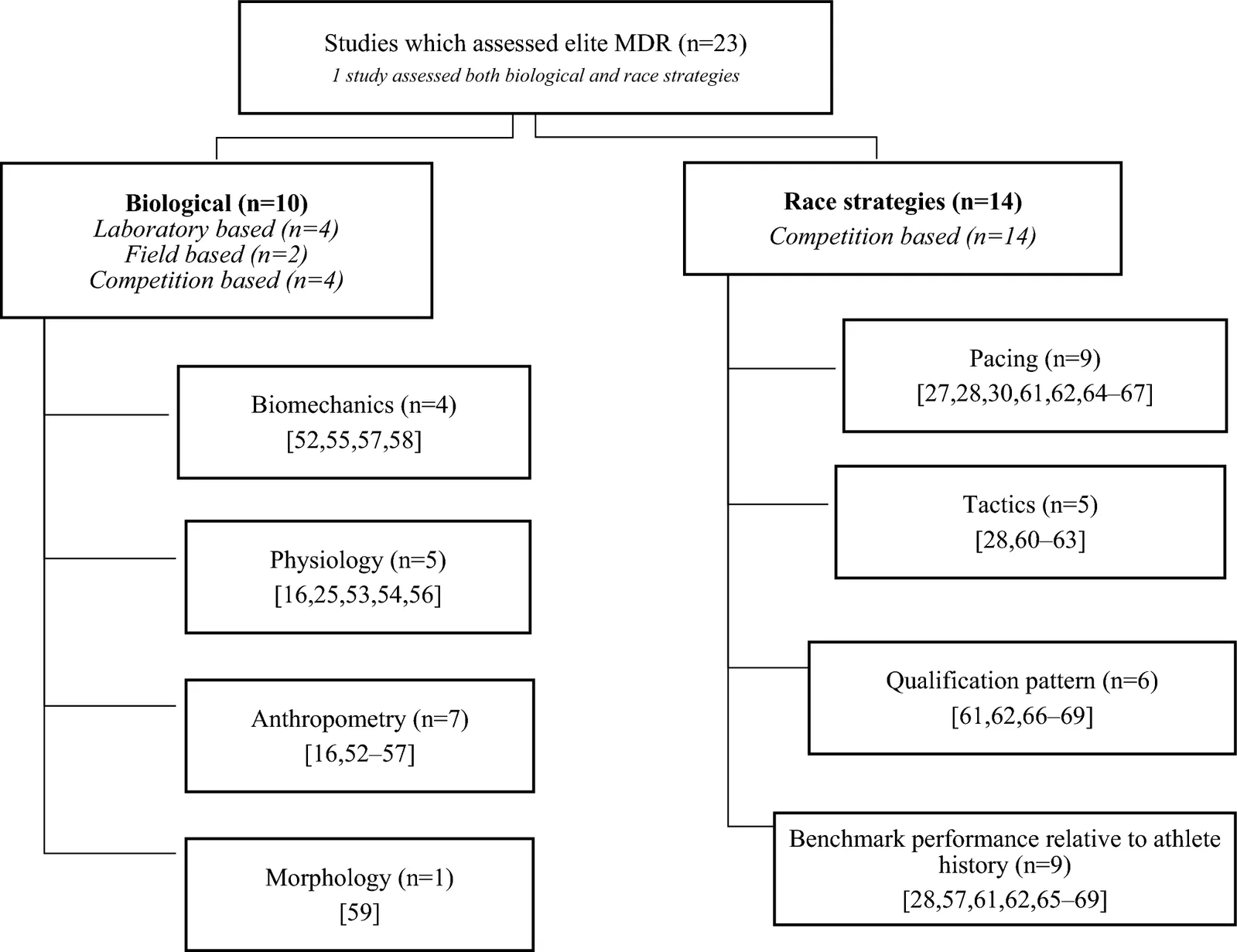
The number of studies (*n*) included that assessed elite middle-distance runners (MDR), grouped by those examining biological or race strategy characteristics and further categorised by specific domains. References provided in [brackets]

Eligible studies which investigated characteristics among elite MDR, including the number of studies relevant to each domain, are provided in Fig. 2.

Table 2 presents the biological and race strategy characteristics investigated in elite MDR across the eligible studies. Characteristics are categorised by their relevant domain, with the number of athletes assessed (or athlete performances for race strategy characteristics only) and the quality score range for the studies investigating each characteristic included. Seven out of the 23 included studies also addressed Aim 2 by investigating whether characteristics could differentiate performance among elite MDR. These studies are presented in Table 3 respective to their relevant domain, with the methodology, main outcomes and quality of the studies reported.

**Table 2:** Summary of biological and race strategy characteristics examined across eligible studies involving elite MDR, categorised by domain and accompanied by the relevant study or studies in [brackets]

Characteristic	Number of athletes assessed				Quality score (%)
	Men’s 800-m	Men’s 1500-m	Women’s 800-m	Women’s 1500-m	
Anthropometry [16, 52–57]					
Body mass [16, 52–57]	1	16	8	1	57–90
Height [16, 52–57]	1	16	8	1	57–90
Body composition [53, 56]	1			1	62–73
Muscle circumferences [53, 56]	1			1	62–73
Bone mineral density [56]				1	73
Physiology [16, 25, 53, 54, 56]					
$\dot{V}$O₂max [53, 54, 56]	1	1		1	57–73
Resting and maximum heart rate [53]	1				62
Blood pressure [53]	1				62
Timed vital capacity [53]	1				62
Maximum breathing capacity [53]	1				62
Oxygen uptake, ventilation, heart rate, true O_2_, blood O_2_ saturation and respiratory quotient [53]	1				62
Haemoglobin and haematocrit [53]	1				62
Running economy [54]		1			57
Maximal aerobic speed [54]		1			57
Lactate threshold [54]		1			57
Post-competition lactate concentration [16]		2			57
Anaerobic speed reserve [25]	4				83
Maximal sprint speed [25]	4				83
Biomechanics [52, 55, 57, 58]					
Spatiotemporal characteristics [52, 55, 57, 58]		13	9		32–87
Joint angles [52, 55, 57, 58]		13	9		32–87
Duration and range of motion of joint angles [52]		5			73
Spring mass characteristics [55]		8			86
Foot strike pattern [57]			8		87
Joint angular velocities [52, 58]		5	1		32–73
Displacement and acceleration of COM [55, 58]		8	1		32–86
Morphology [59]					
Heart morphology [59]	2			1	73
Tactics [28, 60–63]					
Total distance run [28, 60]	26		24		33–82
Theoretical mean speed [28, 60]	26		24		33–82
Theoretical finishing time and race position [28, 60]	26		24		33–82
Changes of race position on bends and straights [28]	24		24		82
Segment changes of race position [28, 61, 62]	24	12	24	12	36–82
Segment extra distance covered on bends [28]	24		24		82
Total changes of positions [28]	24		24		82
Total extra distance covered [28]	24		24		82
Number of surges prior to race kick [63]		36			75
Distance from finish line of final surge and race kick [63]		36			75
Positioning of eventual medallists at initiation of race kick [63]		36			75
Pacing [27, 28, 30, 61, 62, 64–67]					
100-m segment standardised speed scores [28]	24		24		82
100-m segment speed [27, 64]	32^a,b^	42^b^	32^a,b^	32^b^	86–93
Segment speed as a percentage of mean speed [27]	24	36^a^	24^a^	36^a^	93
Differentials between the first half and the second half of the race [27, 30, 65]	62^a,b^	48^a^	32^a^	48^a^	68–93
Pace variability measured through coefficient of variation [27, 64]	32^a,b^	42^b^	32^a,b^	32^b^	86–93
Split times [61, 62, 66, 67]	8	12	8	12	31
Accumulated times [61, 62, 66, 67]	8	12	8	12	31
Qualification patterns [61, 62, 66–69]					
Qualifying times in heats and semi-finals [68, 69]	128^b^	188^b^	125^b^	187^b^	86
Ranking in the heats and semi-finals [69]	120	175	117	141	86
Number of medallists who won heats and/or semi-finals [69]	120	175	117	141	86
Qualification method across heats and semi-finals [69]	120	175	117	141	86
Finishing positions across heats and semi-finals [61, 62, 66, 67]	8	12	8	12	31
Finishing time relative to SB across heats and semi-finals [68]	125	128	119	127	86
Benchmark performance relative to athlete history [28, 57, 61, 62, 65–69]					
Segment speed relative to SB [28]	24		24		82
Occurrence of PB or SB [61, 62, 66, 67]	8	12	8	12	31
Number of gold medallists who achieve PB or SB [69]	15	15	15	12	86
Finishing time relative to SB [68]	125^b^	144^b^	119^b^	143^b^	86
Finishing time relative to PB [57]			8		87
Pacing strategy relative to world ranking [65]	13				68

Number of athletes assessed does not take into consideration repeat athlete measures

*COM* centre of mass, *MDR* middle-distance runners, *PB* personal best, *SB* season best, $\dot{V}$*O₂max* maximal oxygen consumption

^a^Number of athletes not provided; this is an estimate based on the number of championships analysed and the number of athletes normally competing in each final

^b^Represents adjusted athlete number due to repeated measures of the same athlete at the same event across different studies

**Table 3:** Methodology, main outcome and quality of studies evaluating whether biological or race strategy characteristics could differentiate performance among elite MDR

Study	Sample	Testing procedure	Overview of main outcomes	Quality score (%)
Physiology				
Lacour et al. [16]	2 M 1500-m SB: 3:34.91 and 3:35.52	Blood sample obtained within 10 min post-competition	No association between post-competition blood lactate concentration and competition velocity (*p* > 0.05)	57
Biomechanics				
Hanley et al. [55]^a,b^	8 M from the 2017 WC 1500-m final PB: 3:30.93 ± 2.96	Video data (150 Hz) from three high-definition camcorders were digitised Athletes were assessed over one gait cycle during the middle of the home straight on laps 2, 3 and 4	(a) Better finishing positions were found to have coefficients associated with greater overstriding distance, longer foot ahead and behind distances, longer contact times and greater duty factor (*p* < 0.05) (b) Better placed finishers also had greater hip flexion at initial contact, greater knee range of motion from initial contact to midstance, greater hip and knee extension as well as shoulder extension from midstance to toe-off (*p* < 0.05) (c) Better placed finishers also had higher normalised peak horizontal forces, shallower impact angles, greater leg compression, lower leg stiffness, lower vertical stiffness and relative contact velocities (*p* < 0.05)	86
Tactics				
González-Mohíno et al. [28]^a^	24 M and 24 W from the 800-m final at the 2013 and 2017 WC and 2016 OG	Official 100-m segment times from World Athletics website Video data were downloaded from YouTube	(a) Medallists had greater positive changes in intermediate position during the 500–600-m segment compared with non-medallists (*p* = 0.003, *d* = 0.924) (b) Better finishing position was associated with higher positive changes in intermediate position during the 300–400-m (*r* = − 0.42, *p* = 0.003), 500–600-m (*r* = − 0.29, *p* = 0.049) and 700–800-m (*r* = − 0.29, *p* = 0.044) segment (c) Better finishing position was associated with higher total positive changes in intermediate position on bends (*r* = − 0.52, *p* < 0.001) and straights (*r* = − 0.31, *p* = 0.03) (d) Medallists had greater total positive changes in intermediate position on straights compared with non-medallists (*p* = 0.003, *d* = 0.945) (e) Better finishing position was associated with higher total positive changes in intermediate position (*r* = − 0.64, *p* < 0.001) (f) Medallists had greater total positive changes in race position compared with non-medallists (*p* < 0.001, *d* = 1.216)	82
Sandford et al. [63]	36 M medallists from the 1500-m final at the 2000–2017 WC and OG	Video data from YouTube were used	(a) In fast races, with finishing times ≤ 3:34.00, large difference (likely) in the tactical position of the eventual gold (2 ± 1.2) and silver (2.2 ± 1.6) medallists compared with the eventual bronze medallist (4.4 ± 1.3) (b) In medium-paced races, with finishing times 3:34.01–3:41.99, unclear differences in the tactical position of eventual gold (3.6 ± 2.4) and silver medallists (3.1 ± 2.2); however, eventual bronze medallists (5.6 ± 1.6) were in lower positions at the race kick than silver (moderate difference; likely) and gold medallists (moderate difference; possibly)	
Pacing				
González-Mohíno et al. [28]^a^	24 M and 24 W from the 800-m final at the 2013 and 2017 WC and 2016 OG	Official 100-m segment times from World Athletics website Video data were downloaded from YouTube	(a) Finishing position had negative correlations with 100-m segment standardised speed score during the 500–600-m (*r* = − 0.65, *p* < 0.001), 600–700-m (*r* = − 0.81, *p* < 0.001) and 700–800-m (*r* = − 0.73, *p* < 0.001) (b) Medallists had higher (*p* < 0.001) 100-m standardised speed score during the 500–600-m (*d* = 1.553), 600–700-m (*d* = 1.658) and 700–800-m (*d* = 1.268) segments compared with non-medallists (c) Medallists had lower 100-m standardised speed score during the 200–300-m segment compared with non-medallists (*p* = 0.031, *d* = 0.665)	82
Hettinga et al. [27]^a^	24 M and 24 W from the 800-m final and 36 M and 36 W from the 1500-m final at the 2013 and 2017 WC and 2016 OG	Official finishing and split times were obtained from IAAF website, published results and from SEIKO	(a) Faster 100-m segment speed was reported for medallists compared with top 8 athletes during the 500–600-m segment in the 800-m M (*p* < 0.05, d ≥ 0.61) (b) Faster 100-m segment speed was reported for medallists compared with top 8 athletes during the 600–700-m segment in the 800-m W (*p* < 0.05, d ≥ 0.61) (c) Faster 100-m segment speed was reported for medallists compared with top 12 and for top 8 compared with top 12 athletes during the 1400–1500-m segment in the 1500-m M (*p* < 0.05, d ≥ 0.61) (d) Faster 100-m segment speed was reported for medallists compared with top 12 during the 1100–1200-m to 1300–1400-m segment in the 1500-m W (*p* < 0.05, d ≥ 0.61). Faster 100-m segment speed was reported for top 8 compared with top 12 during the 1200–1300-m and 1300–1400-m segment (*p* < 0.05, d ≥ 0.61). Faster 100-m segment speed was reported for medallists compared with top 8 in the 1300–1400-m segment (*p* < 0.05, d ≥ 0.61)	93
Hanley et al. [64]	24 M and 24 W from the 800-m final and 42 M and 32 from the 1500-m final at the 2008 OG and 2013 and 2017 WC, as well as 2016 OG for the 1500-m only	Finishing times and 100-m split times and SB times were collected from IAAF website	(a) Lower cumulative split time was reported during the 700–800-m segment for medallists compared with non-medallists in the 800-m M and 800-m W (*p* < 0.01) (b) No difference in cumulative split time at any 100-m segment was reported between medallists and non-medallists in the 1500-m M and 1500-m W (*p* > 0.05)	86
Qualifying pattern				
Hanley and Hettinga [69]^a^	Finalists in the M and W 800-m and 1500-m at the OG and WC 1999–2017	Finishing position and times were obtained from IAAF website	(a) Better finishing position in the final was associated with better qualifying position in the heat (*p* ≤ 0.002) for all event groups (800-m M: τb = 0.320, 1500-m M: τb = 0.367, 800-m W: τb = 0.400, 1500-m W: τb = 0.344) (b) Better finishing position in the final was associated with better qualifying position in the semi-final (*p* ≤ 0.002) for all event groups (800-m M: τb = 0.229, 1500-m M: τb = 0.412, 800-m W: τb = 0.484, 1500-m W: τb = 0.444) (c) Better finishing time in the final was associated with better ranking in the heats for the 800-m W (τb = 0.213, *p* = 0.001) (d) Better finishing time in the final was associated with better ranking in the semi-final (*p* < 0.001) for the 800-m M (τb = 0.241), the 800-m W (τb = 0.255) and 1500-m W (τb = 0.234)	86
Benchmark performance relative to athlete history				
González-Mohíno et al. [28]^a^	24 M and 24 W from the 800-m final at the 2013 and 2017 WC and 2016 OG	Official 100-m segment times from World Athletics website Video data were downloaded from YouTube	(a) Medallists had higher 100-m segment speed relative to SB speed during the 500–600-m (*p* = 0.016, *d* = 0.749), 600–700-m (*p* < 0.001, *d* = 1.110) and 700–800-m (*p* = 0.007, *d* = 0.843) segments compared with non-medallists (b) Medallists had higher finishing times as a percentage of SB times compared with non-medallists (*p* = 0.042, *d* = 0.622) (c) Better finishing position was associated with higher finishing time as a percentage of SB time (*r* = − 0.48, *p* < 0.001)	82

*CT* competition performance time, *IAAF* International Association of Athletics Federations, *M* men, *MDR* middle-distance runners, *OG* Olympic Games, *PB* personal best performance time, *SB* season best performance time, *W* women, *WC* World Championships

^a^For these studies, only characteristics which showed significant associations or capacity to differentiate performance were provided in overview of main outcome section

^b^Magnitude or strength of association was not provided as only coefficients were provided

**Fig. 3:**
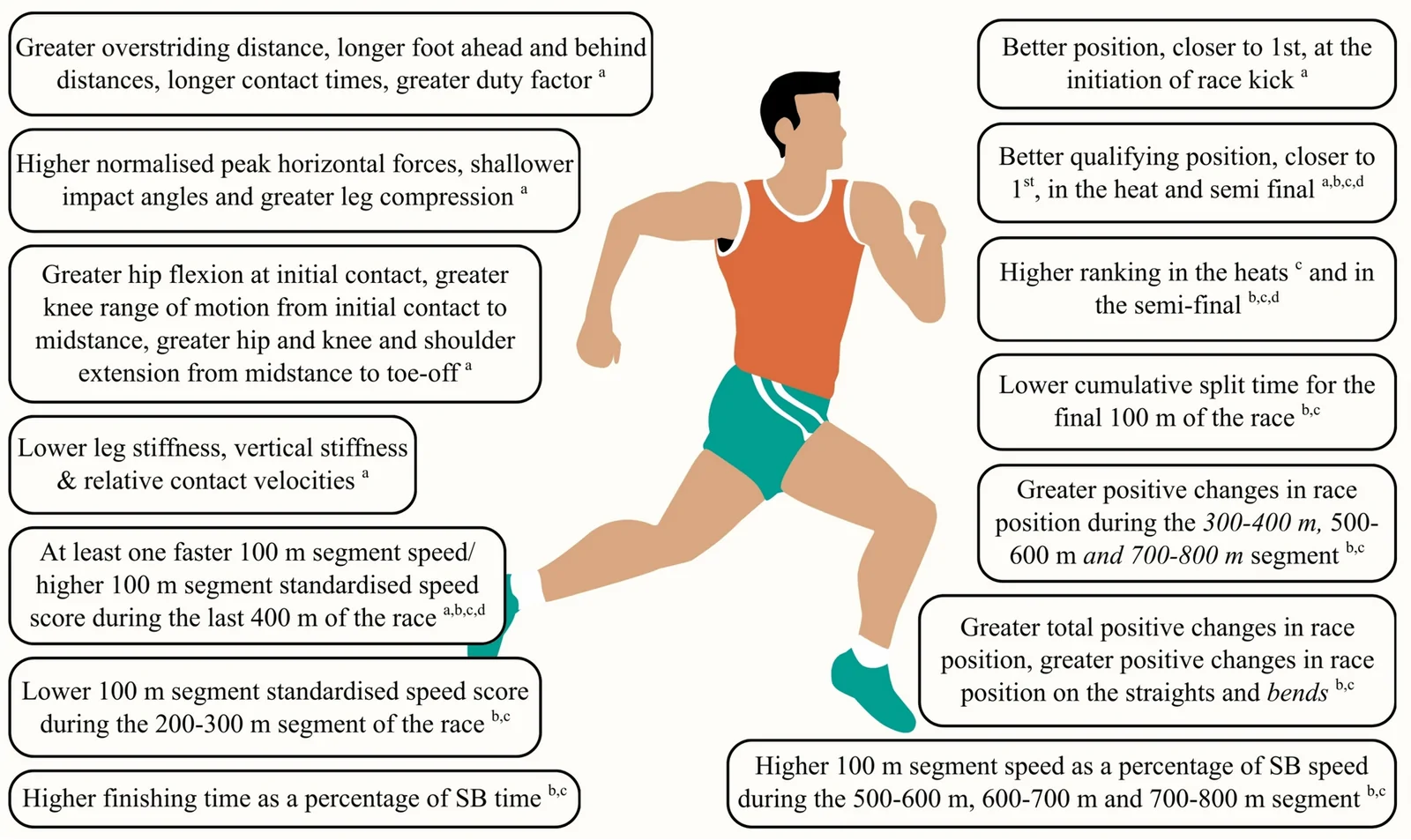
Biological and race strategy characteristics associated with better performance in elite middle-distance running. Characteristics differentiated performance among ^a^ male 1500-m runners, ^b^ male 800-m runners, ^c^ female 800-m runners, ^d^ female 1500-m runners. *Italics* are used to represent mixed evidence, with differentiation found in some instances but not all. *SB* season best performance time

A summary of biological and race strategy characteristics associated with faster finishing times or better finishing positions among elite MDR is provided in Fig. 3.

##### Anthropometry

The majority of studies reporting anthropometric characteristics only reported body mass and height as standard practice for reporting participant demographics [16, 52, 54, 55, 57]. Further, the other two studies investigated the anthropometric profile of elite MDR, however, were single athlete case studies [53, 56]. For elite male 1500-m runners, a weighted body mass and height were reported as 66.4 kg and 1.80 m, respectively [16, 52, 54, 55]. For elite female 800-m runners, body mass and height were reported as 61 ± 6 kg and 1.73 ± 0.06 m, respectively [57]. Measurement methods were not consistently reported across studies.

##### Physiology

Studies reporting physiological characteristics included single-athlete case studies [53, 54, 56], two [16] and four eligible athletes [25]. One of these studies found no association between post-competition blood lactate concentration and performance [16] (Table 3).

##### Biomechanics

Biomechanical characteristics were determined through competition video analysis in all studies [52, 55, 57, 58]. Two studies examined elite female MDR. Hanley et al. analysed eight finalists in the women’s 800-m at the 2017 World Championships [57], whereas Skof and Stuhec assessed one female 800-m runner at a European Athletic Association meeting [58]. The single-athlete study provided insufficient methodological detail to warrant comparison [58]. Hanley et al. [57] reported characteristics including step length (1.95–1.97 m), step frequency (3.28–3.52 Hz), contact time (0.151–0.159 s), knee angle at initial contact (23°–28°) and ankle angle at initial contact (110°–113°) at a running velocity of 6.46–6.85 m/s. Further, athletes were analysed on both laps with faster speeds achieved on lap 2 through increases in cadence (*p* = 0.012), rather than step length, which was accompanied by shorter flight (*p* = 0.016) and swing times (*p* = 0.016), greater flexion of the knee (*p* = 0.008) and greater plantarflexion of the ankle at initial contact (*p* = 0.039) [57].

Two studies investigated elite male MDR, with finalists in the men’s 1500-m at the World Championships in 2005 [52] and 2017 [55] analysed. Hanley et al. [55] reported slightly higher step frequency (3.34 Hz vs 3.2 Hz) and slightly shorter contact times (0.147 s vs 0.154 s) in comparison with Leskinen et al. [52]. Hanley et al. [55] also reported mean step length of 2.16 m, hip angle at contact of 152°, knee angle at contact of 154° and vertical stiffness of 48.5 kN/m for eight finalists. Further, faster running speeds were associated with increased step frequency and reduced contact time [55]. Leskinen et al. [52] also reported mean minimum knee angle during stance of 138°, hip angle during contralateral midstance of 132° and knee extension velocity during the stance phase of 5.6 rad/s for five finalists. Additionally, Hanley et al. [55] identified various biomechanical characteristics associated with finishing position (Table 3).

##### Morphology

One longitudinal study assessed heart morphology in two elite male and one elite female MDR over 9 years [59]. Within athletes, increases in left ventricular internal diameter in diastole (LVIDd) were associated with performance improvements in the elite male 800-m runners (*p* < 0.008), and decreases in end-diastolic posterior wall thickness (PWT) were associated with improved performance across the sample (*p* < 0.05) [59].

##### Pacing

Five studies reported pacing characteristics from a single benchmark event [30, 61, 62, 66, 67], whereas four studies analysed multiple benchmark events [27, 28, 64, 65]. Of these, three studies [27, 28, 64] investigated whether pacing characteristics differentiated performance (Table 3).

In the men’s and women’s 800-m finals, 100-m segment speed across four benchmark events demonstrated a seahorse-shaped pacing profile [27, 64]. Speeds were fastest over the first 200 m, declined markedly from 200 to 300 m, remained relatively stable until 500 m, increased from 500 to 600 m and decreased over the final 200 m [27, 64]. In the men’s and women’s 1500-m finals, 100-m segment speed across four benchmark events demonstrated a J-shaped pacing profile [64]. Athletes progressively increased speed between 500 and 700 m, continuing this acceleration until approximately 1300 m, after which speed was either maintained or slightly reduced over the final 200 m [64].

Pace variability has been examined across four benchmark events [27, 64]. Pace variability based on the coefficient of variation of all 100-m segment speeds was on average 4.6–9.7% for elite MDR [27, 64]. Pace variability was higher in the 1500-m compared with the 800-m finals [27, 64].

Pacing differentials between the first half and the second half of the race across 13 championships for the men’s 800-m [27, 30, 65] have been examined. Between 2000 and 2009, races typically demonstrated a negative pacing profile, characterised by faster second halves [30, 65]. From 2011 to 2017, this shifted to positive pacing profiles, with faster first halves [27, 65]. Pacing differentials were also reported across four championships for other middle-distance events [27, 30]. In the women’s 800-m, races demonstrated a positive pacing profile at benchmark events from 2008 onwards [27, 30], whereas in the men’s and women’s 1500-m, there was a consistent adoption of a negative pacing profile [27, 30].

##### Tactics

Three studies reported tactical characteristics from a single benchmark event [60–62], whereas two studies analysed multiple benchmark events [28, 63]. Of these, two studies [28, 63] examined whether tactical characteristics differentiated performance (Table 3). In men’s 1500-m championship races, fast-paced races (≤ 3:34.00) were characterised by fewer surges (1) compared with medium-paced races (< 3:42.00; 4 surges) [63]. The race kick occurred at ~ 240 m from the finish in both race types, whereas the final surge occurred earlier in fast races (704 m) than in medium-paced races (427 m) [63].

##### Qualification Patterns

Four studies reported qualification patterns for one benchmark event [61, 62, 66, 67] and two studies analysed multiple benchmark events [68, 69]. Of these, one study examined whether qualification patterns differentiated performance at benchmark events [69] (Table 3).

Most medallists won at least one preliminary round (gold: 89%; silver: 79%; bronze: 51%) and 70% of gold medallists won both qualification rounds, compared with 36% of silver and 19% of bronze medallists [69]. Qualification times in the heats and semi-finals were not different between medallists and non-medallists or between medal categories in any MDR event [69]. No male medallists and four female medallists were ranked first in both the heats and semi-final [69]. On average, medallists were ranked between 3rd and 62nd across heats and semi-finals, indicating substantial variability in preliminary-round rankings [69]. In the women’s 800-m and men’s 1500-m, medallists ran faster in the final compared with the semi-final (*p* < 0.001) [69]. In contrast, top-8 athletes in the 800-m and top-12 athletes in the 1500-m maintained or slowed their pace in the final compared with their semi-final [69]. Qualification times in the heats and semi-finals were significantly slower than SB times for medallists and top-8 athletes [68].

##### Benchmark Performance Relative to Athlete History

Five studies reported benchmark performance relative to athlete history at a single competition [57, 61, 62, 66, 67], whereas four studies analysed multiple benchmark events [28, 65, 68, 69]. Of these, one study examined whether benchmark performance relative to athlete history could differentiate elite MDR [28] (Table 3).

In 800-m benchmark event finals, medallists were able to exceed their SB pace in the 500–600-m and 600–700-m segments [28]. Further, in the men’s 800m, medallists ran times similar to their SB (*p* > 0.05, *d* = 0.27), whereas top-8 athletes ran slower than their SB (*p* < 0.001, *d* = 1.18) [68]. In the women’s 800-m, medallists ran faster than their SB (*p* < 0.05, *d* = 0.39), while no difference between finishing time and SB was observed for top-8 athletes (*p* > 0.05, *d* = 0.26) [68]. Of the 15 Olympic and World Championship gold medallists, six 800-m men and nine 800-m women achieved PB or SB times in the final [69]. Eventual male 800-m champions who had higher, better world rankings prior to the championship were more likely to adopt a positive pacing strategy (*r* = 0.94, 0.84–0.98; extremely large, most likely) whereas races won by athletes who had lower, worse world rankings tended to adopt a negative pacing approach [65].

Finishing times were slower than SB times for medallists in the men’s (*p* < 0.001, *d* = 0.86) and women’s (*p* < 0.05, *d* = 0.56), and top-8 athletes in the men’s (*p* < 0.001, *d* = 1.05) and women’s (*p* < 0.001, *d* = 0.76) 5000-m [68]. Of the 15 Olympic and World Championship gold medallists, three women and no men in the 1500-m achieved PB or SB times in the final [69].

#### Discussion for Middle-Distance Running

##### Anthropometry

Studies reporting anthropometric characteristics included standard participant demographic reporting [16, 52, 54, 55, 57] and single-athlete case studies [53, 56], which limited the ability to draw conclusions about the broader elite middle-distance running population. The reported body mass and height for elite male and female MDR were slightly higher than values reported for highly trained and near-elite runners [15, 18, 70], which may reflect difference in performance standards. However, these differences could also have been influenced by age, with elite athletes aged ~ 26 to 27 years [16, 52–57] compared with 19.7–22.1 years in the highly trained and near-elite runners [15, 18, 70]. In addition, the reliability of the anthropometric data for elite athletes was uncertain because several studies did not specify measurement methods [16, 52, 54] or collated data from external sources [55, 57]. Given these limitations, current evidence is insufficient to characterise the anthropometric profile of elite middle-distance runners beyond basic reporting of body mass and height.

There is currently no evidence investigating the capacity of anthropometrical characteristics to differentiate performance among elite MDR. Findings from lower-standard MDR show relationships between better performance and lower skinfold thickness [23, 70], lower body fat percentage [70], greater total lean body mass [21] and greater lower leg to upper leg mass [21]. Based on this evidence [21, 23, 70], there is good theoretical grounding to investigate whether these anthropometric characteristics can differentiate performance among elite MDR. However, elite MDR are often either anaerobic or aerobic specialists, both of which require distinct training demands that can lead to differences in anthropometry unrelated to performance capacity [71].

##### Physiology

The small sample sizes and predominance of single-athlete case studies limited the ability to draw conclusions about physiological characteristics in the broader elite middle-distance running population. One of these studies investigated whether physiological characteristics could differentiate performance among elite MDR [16]. Post-competition blood lactate had no association with competition velocity for two male 1500-m runners measured across six competitions [16]. However, given the small sample size and repeated athlete measures, current evidence is insufficient to determine whether post-competition blood lactate differentiates performance among elite MDR.

Findings among highly trained and near-elite MDR revealed associations between better performance and greater end-exercise blood lactate accumulation [15], greater maximal capacity of the muscle to extract oxygen from the blood [15], greater velocity at $\dot{V}$O₂max [14, 18, 72], greater $\dot{V}$O₂max (absolute and relative) [18], greater fraction utilisation of $\dot{V}$O₂max at 5.42 m/s [72], greater post-competition blood lactate concentrations [16] and greater speed at 4 mmol·L blood lactate [72]. Further, greater maximal sprint speed and anaerobic speed reserve were associated with faster 800-m performance times among near-elite male runners [25]. Based on this evidence [14, 15, 18, 25, 72], there is good theoretical grounding to investigate whether physiological characteristics can differentiate performance among elite MDR. However, it is possible that once a certain performance threshold is reached, associations between physiology characteristics and performance may no longer exist due to the low variability between elite athletes.

##### Biomechanics

Studies investigating biomechanical characteristics in elite female MDR are limited [57, 58]. Comparison between studies was not possible, as Skof and Stuhec’s study [58] provided insufficient methodological information. Further, Skof and Stuhec’s study [58] was of low quality and involved the assessment of only one athlete, making it difficult to draw any conclusions about the broader elite middle-distance running population. The most informative biomechanical data were from Hanley et al., who analysed eight female 800-m finalists at the World Championships 2017 [57]. Increased cadence, rather than step length, drove faster second lap speeds, indicating that cadence-related mechanics underpin sprint finish performance in elite female 800-m runners [57]. It may be important for elite female 800-m runners to develop the neural adaptations required for higher cadence to achieve the finishing speeds required at benchmark events. Hanley et al.’s study provides valuable insight into the biomechanics of elite female middle-distance runners [57]; however, further research is needed to advance this understanding.

The available biomechanical evidence provides some insight into the typical running mechanics of elite male MDR [52, 55]. These studies both reported contact time and step frequency, with slightly shorter contact times and higher step frequencies reported by Hanley et al. [55]. These small differences are likely attributable to the higher running speeds in Hanley et al.’s study [55] (approximately 6.4–7.7 m/s) compared with the average speed reported by Leskinen et al. [52] (6.5 m/s). This interpretation is in alignment with previous literature which demonstrates that increases in speed are achieved by decreasing contact time and increasing step frequency, especially at faster speeds [55, 73], underscoring the importance of interpreting these characteristics relative to running speed. Faster running speeds were achieved through increased cadence and reduced contact time, rather than changes in step length, highlighting the importance of cadence modulation in producing the high speeds required during key phases of the men’s 1500-m [55]. With only two studies and 13 athletes examined, and limited overlap in reported variables, larger investigations are needed to establish whether these findings reflect elite male middle-distance running more broadly.

Additionally, one of these studies investigated whether biomechanical characteristics could differentiate performance among elite MDR [55]. Several spatiotemporal, joint angle and spring mass characteristics were associated with a better finishing position (*p* < 0.05) in the 2017 World Championship men’s 1500-m final [55]. The findings suggest that a technique favouring greater top speeds, rather than economy, may be capable of differentiating performance at benchmark events [55]. Longer ground contacts, relatively ‘softer’ spring-mass characteristics (more compliant spring mechanics) and greater knee flexion were associated with better finishing positions [55]. This technique may enhance horizontal propulsive force production, supporting the fast endspurt speeds required for success in elite middle-distance running races [27, 28, 64]. However, some methodological limitations of this study should be considered when interpreting the strength of these findings; spring mass characteristics were estimated using temporal measures and biomechanical characteristics were measured based on a single gait cycle on each lap [55]. Further research is needed to determine whether the identified biomechanical characteristics [55] can consistently differentiate performance among elite MDR, particularly across sexes and event distances.

Aligning with findings in the elite population [55], no differences were found between near-elite and trained 1500-m runners for step frequency, vertical displacement and step length [73]. Further, no differences were found between elite and highly trained 1500-m runners for step frequency [52]. Together, these findings suggest that commonly reported spatiotemporal characteristics may not be capable of differentiating performance among elite MDR. In contrast with findings in the elite population in which better placed finishers had longer contact times [55], no difference in contact times was found between elite and highly trained runners [52]. This discrepancy may indicate that contact time behaves differently in championship racing, where slightly longer contact times could facilitate greater propulsive forces during the sprint finish.

Consistent with findings in the elite population [55], Trowell et al. [20] found that faster highly trained 1500-m runners had greater knee flexion during stance (*p* = 0.055), which the authors proposed reflected greater tendon compliance. In contrast, elite runners had smaller knee extension angular velocities than highly trained runners, likely due to their reduced and shorter duration knee flexion in the loading phase of contact, indicative of greater lower limb stiffness [52]. Further supporting this, near-elite runners exhibited greater peak vertical forces, higher leg and vertical stiffness and larger impact angles compared with trained runners [73]. Although these findings appear contradictory [20, 52, 55, 73], they likely reflect differences in the performance standard of athletes investigated. Compared with lower standard runners, greater spring-mass stiffness may be important for high-performing runners to enhance running efficiency [52, 73]. However, among high-performing runners, relatively more compliant spring-mass behaviour may be advantageous for more favourable force–length–velocity relationships which enable greater force production for faster endspurt speeds [20, 55]. Overall, this suggests that optimal spring-mass characteristics in middle-distance running are context dependent, rather than uniformly favouring greater stiffness or compliance.

In addition, other biomechanical characteristics have shown the capacity to differentiate performance among lower performing MDR [20, 52, 73]. Specifically, elite male 1500-m runners were characterised by more efficient functioning of the hip joint and the knee extensors compared with highly trained runners [52]. Further research should assess whether these characteristics are associated with performance among elite MDR.

##### Morphology

Evidence on heart morphology in elite MDR is very limited [59]. A small longitudinal study of elite MDR found that heart morphology, specifically increases in LVIDd and decreases in PWT, may be associated with improvements in individual performance [59]. It is proposed that an increase in left ventricular internal diameter could be important for elite middle-distance running performance as it allows for a more rapid start of aerobic metabolism and can help to speed up removal of by-products derived from metabolism [74, 75]. However, cross-sectional data from highly trained and near-elite MDR found no association between LVIDd or PWT and 800-m or 1500-m race times [74]. Heart morphology may be useful for monitoring individual changes in elite athletes; however, current evidence does not support their use to differentiate performance between athletes. Studies including larger elite samples are needed to assess whether heart morphology can differentiate performance among elite MDR.

##### Pacing

Current evidence provides insight into pacing strategies used by elite MDR at benchmark events. Specifically, three studies investigated whether pacing characteristics differentiated performance at multiple benchmark events, helping to provide an understanding of recurring pacing behaviours [27, 28, 64].

The observed seahorse pacing profile reflects the tactical and physical demands of elite 800-m competition. The aggressive opening 200 m likely facilitates optimal positioning [27, 64]. The ability to increase speed from 500 m despite accumulated fatigue appears particularly important, supporting the notion that end-race speed capacity is a key component of elite 800-m racing [27, 64]. Studies investigating associations between pacing characteristics and performance consistently found that athletes with superior finishing positions were faster during at least one 100-m segment after the 500 m mark in 800-m benchmark finals [27, 28, 64]. These findings indicate that the ability to increase and then maintain race speed in the latter stages of the race may be a key performance determinant of elite 800-m racing.

The J-shaped pacing profile observed in elite 1500-m finals suggests a progressively intensifying race structure, a pattern that indicates that performance may depend less on a single abrupt sprint and more on the capacity to progressively accelerate and sustain high running speeds during the closing stages. This profile supports the importance of speed endurance in elite 1500-m racing. Medallists had faster 100-m segment speed compared with non-medallists during the latter stages of the race [27], whereas cumulative 100-m split time did not differ between groups [64]. The apparent discrepancy between studies is likely attributable to the different pacing metrics assessed. Cumulative split time reflects accumulated race time [64], whereas 100-m segment speed captures discrete velocity changes within specific race segments [27]. As segment speed differences emerged primarily in the closing stages, cumulative split times at these points would closely reflect overall finishing time, which did not differ between groups. These findings suggest that 100-m segment-based speed in the latter phases of the race may be able to differentiate performance in 1500-m benchmark finals [27], reinforcing the importance of speed maintenance under fatigue.

The observed magnitude of pace variability suggests that elite middle-distance performance occurs under fluctuating speed conditions rather than even pacing [27, 64]. The consistently greater variability in the 1500-m compared with the 800-m may reflect the more tactical nature of 1500-m racing [27, 64]. These findings highlight the importance for elite MDR to be able to tolerate and respond to pace fluctuations [27, 64].

Contemporary championship 800-m racing appears to be characterised by predominantly positive pacing profiles [27, 30, 65], indicating the importance of a fast opening lap. This may place greater emphasis on early race positioning and the capacity to achieve and tolerate high initial speeds. In contrast, the 1500-m is consistently characterised by a negative pacing profile, reflecting a progressively intensifying race [27, 30]. These findings suggest that preparation for the 1500-m should prioritise the capacity to progressively increase and sustain speed during the second half of the race.

##### Tactics

Current evidence provides some insight into tactical strategies used by elite MDR at benchmark events [28, 60–63]. Specifically, two studies analysed multiple benchmark events, providing a more robust understanding of recurring tactical behaviours and their association with performance outcomes [28, 63].

González-Mohíno et al. [28] found that medallists compared with non-medallists had greater positive changes in position throughout the race, especially on straights and during the 500–600-m segment, at benchmark 800-m events [28]. This demonstrates better tactical decision making and the importance of being able to accelerate with approximately 300 m remaining for medal contention. However, total extra distance run was not associated with finishing position and did not differentiate medallists from non-medallists [28]. Further, better placed athletes were characterised by greater positional gains on both straights and bends [28]. These findings highlight that covering extra distance on bends can be unavoidable and does not necessarily compromise finishing position.

Sandford et al. [63] found that gold and silver medallists were in better positions, closer to first, at the initiation of race kick compared with bronze medallists in the men’s 1500-m final at benchmark events [63]. This suggests that positioning immediately before the decisive kick is an important tactical factor for medal outcomes. Research into whether race position at initiation of race kick can differentiate performance among medallists and non-medallists and in other elite men’s and women’s middle-distance running races is therefore of interest. These findings could help inform tactics for coaches and runners targeting elite endurance track events. Further, this study showed that the tactical structure of men’s 1500-m benchmark events varies according to overall pace. Fast-paced finals involved fewer kicks whereas medium-paced finals were characterised by more frequent changes in pace, and it is important that elite MDR are physically and tactically prepared for both types of races. Race kick occurred at a similar point no matter whether it was a fast- or medium-paced final (~ 240 m from the finish). This suggests that elite MDR should repeatedly rehearse initiating and sustaining accelerations from 250 m out under fatigue and prioritise securing a competitive tactical position before this point in races.

##### Qualification Pattern

Current evidence provides insight into qualification patterns used by elite MDR at benchmark events [61, 62, 66–69]. Specifically, Hanley and Hettinga [69] investigated qualification patterns at 15 benchmark events, providing a strong understanding of recurring qualification patterns and their association with performance outcomes. Qualifying position, rather than qualifying time, in the heats and semi-final appears to be a meaningful indicator of finishing position in the final, with athletes capable of finishing at the front of heats and semi-finals more likely to win a medal [69]. Further, gold medallists were more likely to win both qualification rounds, suggesting that dominance across rounds may differentiate eventual champions from other finalists [69]. The absence of differences in qualifying times between medallists and non-medallists suggests that medallists are able to control their races and secure better qualifying positions without running significantly faster than their competitors [69]. There were limited associations between heat rankings and finishing times; this, combined with medallists’ wide variability in rankings in the heats and semi-final, suggests that athletes prioritise qualification over maximal effort in the heats. In contrast, semi-final rankings were associated with finishing time in most middle-distance running events, indicating that performance in later rounds may be more indicative of championship success.

The ability to run faster in the final compared with the semi-final seems to be important for medal contention at benchmark events [69]. In contrast, non-medallists were more likely to maintain or slow their pace in the final [69], suggesting that these athletes may have run closer to their maximum capacity in the semi-final to qualify, limiting their ability to improve their performance in the final race. Coaches should develop athletes’ physical resilience to compete across rounds while also refining their tactical ability to manage effort strategically throughout the championship. Finalists in elite middle-distance running events are generally able to qualify while performing below their SB standard [68], potentially reflecting deliberate energy conservation across rounds. The ability to qualify without reaching maximal performance may therefore represent an important characteristic for progressing to and succeeding in benchmark event finals.

##### Benchmark Performance Relative to Athlete History

Current evidence provides insight into benchmark performance relative to athlete history for elite MDR [28, 57, 61, 62, 65–69], particularly from studies examining multiple benchmark events [28, 65, 68, 69]. González-Mohíno et al. [28] highlighted the importance of being able to perform close to SB speed in the 800-m. Medallists and better placed finishers were able to perform closer to their SB [28]. Further, medallists were able to exceed SB relative speeds during the final 300 m, suggesting the ability to perform at or above SB speed in the decisive stages of championship races may be important for medal contention [28]. Consistent with these findings, medallists ran closer to their SB times compared with non-medallists [68] and a substantial proportion of Olympic and World Champions achieved a PB or SB [69] in 800-m benchmark finals. Collectively, these findings suggest that being able to closely reproduce or exceed SB performance during the final may be important for medal contention in the 800-m. Additionally, in the men’s 800-m, the eventual gold medallist’s pre-championship world ranking appears to influence the pacing approach in the final [65]. Races won by higher ranked athletes were more likely to adopt a positive pacing strategy, whereas victories by lower ranked athletes tended to occur in races characterised by negative pacing [65]. This suggests that higher-ranked athletes may benefit from imposing a faster overall pace from the start of the race to reduce the likelihood of victory relying on a large pack sprint finish.

In 1500-m benchmark finals, both medallists and non-medallists were typically slower than their SB times [68] and very few champions achieved PB or SB in the final [69]. These findings indicate that tactical execution and race dynamics may play a more influential role in determining race outcomes than achieving PB or SB times in the 1500-m.

### Long-Distance Running

#### Results for Long-Distance Running

**Fig. 4:**
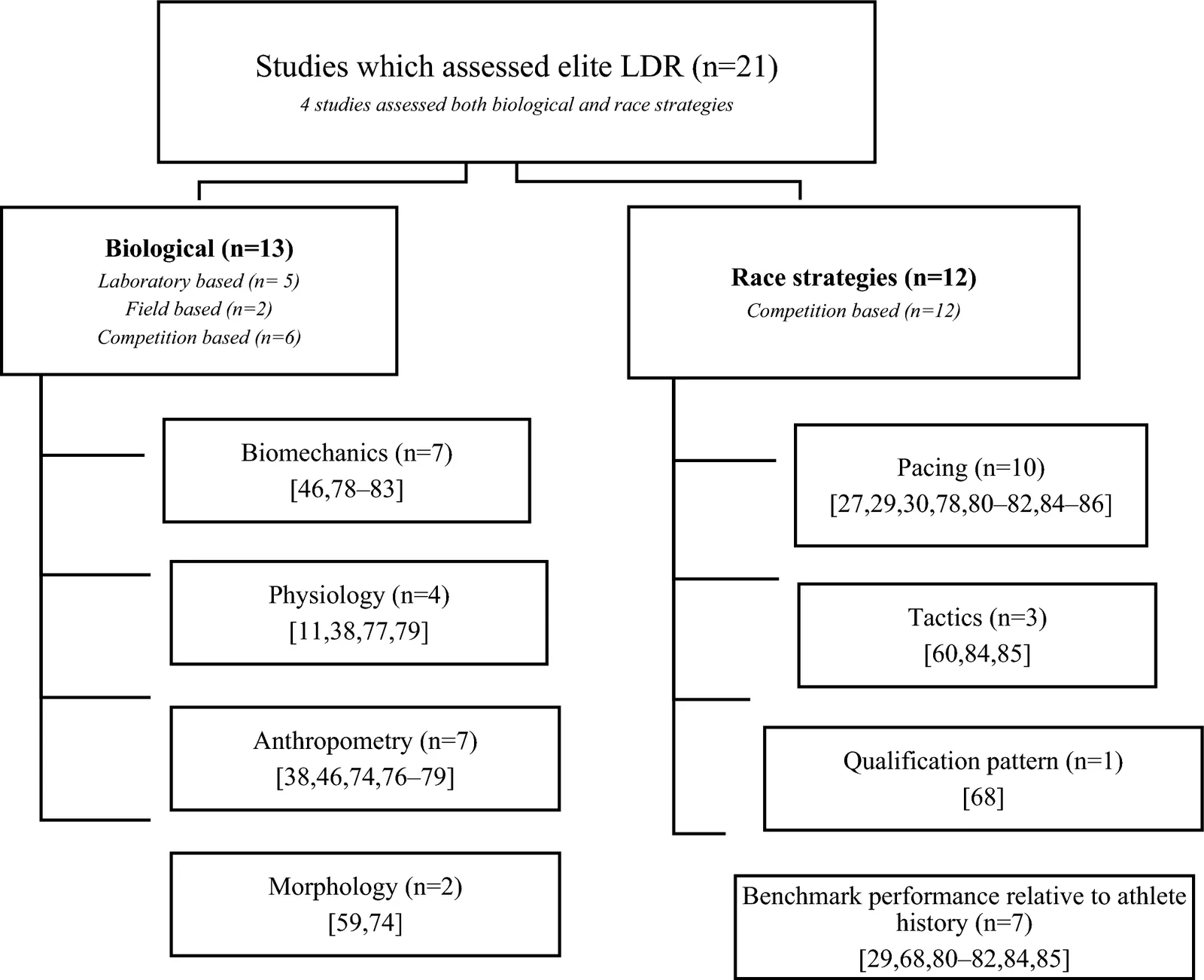
The number of studies (*n*) included specific to elite long-distance runners (LDR), grouped by those examining biological or race strategy characteristics and further categorised by specific domains. References provided in [brackets]

Eligible studies which investigated characteristics among elite LDR, including the number of studies relevant to each domain, are provided in Fig. 4.

Table 4 presents the biological and race strategy characteristics investigated in elite LDR across the eligible studies. Characteristics are categorised by their relevant domain, with the number of athletes assessed (or athlete performances for race strategy characteristics only) and the quality score range for the studies reporting each characteristic included. Five out of the 21 included studies also addressed Aim 2 by investigating whether characteristics could differentiate performance among elite LDR. These studies are presented in Table 5 respective to their relevant domain, with the methodology, main outcomes and quality of the studies reported.

**Table 4:** Summary of biological and race strategy characteristics examined across eligible studies involving elite LDR, categorised by domain and accompanied by the relevant study or studies in [brackets]

Characteristic	Number of athletes assessed						Quality score (%)
	Men’s 3000-m SC	Men’s 5000-m	Men’s 10,000-m	Women’s 3000-m SC	Women’s 5000-m	Women’s 10,000-m	
*Anthropometry* [38, 46, 74, 76–79]							
Body surface area [74]					2		67
Body mass [38, 76–79]	13		33			16	47–93
Height [46, 76–79]	13		33	13		16	47–96
Limb length [76, 79]			25			16	83–93
Limb circumference [76, 79]			25			16	83–93
*Physiology* [11, 38, 77, 79]							
Heart rate max [38, 77]	13		5				80–93
$\dot{V}$O₂max [38, 77, 79]	13		14				80–93
Maximal arteriovenous oxygen difference [77]							80
Cardiac output [77]			5				80
Stroke volume [77]			5				80
Energy cost of running [38]	13						93
Running economy [11, 79]			10				75–87
100-m and 400-m sprint times [11]			1				75
Standing broad jump [11]			1				75
*Biomechanics* [46, 78–83]							
Spatiotemporal characteristics [46, 78–80, 82, 83]	13		20	14		8	38–97
Take-off angle [46, 83]	13			14			60–96
Clearance height [46] or clearance ratio [83]	13			14			60–96
Effectiveness index of mechanical energy utilisation to running velocity [78]			3				47
Mean power [78]			3				47
Joint angles [78, 80, 82, 83]			11	1		8	38–60
Joint angular velocity [78, 83]			3				47–60
Footstrike pattern [81]			12				97
Horizontal and vertical velocity of COM [83]				1			60
Height and trajectory of COM [83]				1			60
*Morphology* [59, 74]							
Heart morphology [59, 74]		1			3		67–73
*Tactics* [60, 84, 85]							
Total distance run [60]		2					33
Theoretical mean speed [60]		2					33
Theoretical finishing time and race position [60]		2					33
Segment race position [84, 85]		14			14		31
*Pacing* [27, 29, 30, 78, 80–82, 84–86]							
Split times [29, 78]		73	83		69	80	47–93
Accumulated times [78, 80, 82, 84, 85]		14	11		14	8	31–47
Segment speed [27, 30, 78, 86]	28	59^a^	56^a^	30	60^a^	53^a^	47–100
Pace variability measured through coefficient of variation [27, 30, 86]	28	59^a^	53^a^	30	60^a^	53^a^	79–100
Differentials between the first half and the second half of the race [27, 30]		59^a^	53^a^		60^a^	53^a^	79–93
Largest percentage speed reduction throughout the race [30]		14	8		15	8	79
Endspurt speed as a percentage relative to runner’s speed in the last quarter of the race [30]		14	8		15	8	79
Segment speed as a percentage of mean speed [27]		45^a^	45^a^		45^a^	45^a^	93
*Qualification patterns* [68]							
Qualifying times across the heats [68]		133			128		86
Qualifying times across the heats relative to SB [68]		133			128		86
*Benchmark performance relative to athlete history* [29, 68, 80–82, 84, 85]							
Finishing time relative to 32-month best time [29]		73	80		69	80	93
Occurrence of PB or SB [80, 82, 84, 85]		14	8		14	8	31–32
Number of athletes who achieved a PB [81]			12				97
Finishing time relative to SB [68, 80, 82]		133	8		128	8	32–86
Finishing time relative to PB [80, 82]			8			8	32
Finishing position relative to PB and SB ranking [80, 82]			8			8	32

Number of athletes assessed does not take into consideration repeat athlete measures

*COM* centre of mass, *LDR* long-distance runners, *PB* personal best, *SB* season best, *SC* steeplechase, $\dot{V}$*O₂max* maximal oxygen consumption

^a^Number of athletes not provided; this is an estimate based on the number of championships analysed and the number of athletes normally competing in each final

**Table 5:** Methodology, main outcome and quality of studies evaluating whether biological or race strategy characteristics could differentiate performance among elite LDR

Study	Sample	Testing procedure	Overview of main outcomes	Quality score
*Anthropometry*				
Dessalew et al. [76]^a^	16 M and 16 W 10,000-m runners M: 27 min 11 s W: 31 min 07 s	Weight was measured on a scale and height on a stadiometer Limb length and circumference were measured using calibrated measuring tape	a) Faster finishing times were associated with lower body mass for men (*r* = 0.585, *p* = 0.017) and women (*r* = 0.517, *p* = 0.040) b) Faster finishing times were associated with smaller arm circumferences for men (*r* = 0.590, *p* = 0.016) and women (*r* = 0.539, *p* = 0.031) c) Faster finishing times were associated with smaller calf circumferences for men (*r* = 0.498, *p* = 0.050) and women (*r* = 0.506, *p* = 0.046) d) Faster finishing times were associated with smaller height for men (*r* = 0.729, *p* = 0.002) e) Faster finishing times were associated with shorter thigh lengths for men (*r* = 0.584, *p* = 0.018) f) Faster finishing times were associated with smaller thigh circumferences for men (*r* = 0.620, *p* = 0.010) g) Height was found to have positive prediction effect on finishing time for men (*R*^2^ = 0.582, *p* < 0.05) h) Arm circumference was found to have positive prediction effect on finishing time for women (R^2^ = 0.926, *p* < 0.05) However, none of the above anthropometric characteristics showed a partial correlation with performance in the absence of their corresponding covariants for men or women	93%
*Physiology*				
Tartaruga et al. [38]	13 M 3000-m SC runners PB: 8 min 29 s	Incremental treadmill test to exhaustion 6-min submaximal running test at 70% of $\dot{V}$O₂max	Recent best performance, measured by IAAF points, did not correlate with $\dot{V}$O₂max with or without the application of specific allometric exponents (*p* > 0.05)	93%
Santos-Concejero et al. [79]	9 M 10,000-m runners PB: 27 min 42 s ± 48 s	6-min submaximal treadmill running test at 17, 19 and 21 km/h	Recent 10,000-m performance was not correlated with the average running economy among elite Eritrean runners (*p* > 0.05)	83%
*Pacing*				
Hanley and Williams [86]	28 M and 30 W 3000-m SC runners from the 2008 and 2016 OG finals	Official finishing and split times were obtained from results documents	a) Top-8 athletes had lower cumulative split time initially at lap 4 compared with non–top-8 athletes in the men’s 3000-m steeplechase (*p* < 0.05) b) Top-8 athletes had lower cumulative split time initially at lap 2 compared with non–top-8 athletes in the women’s 3000-m steeplechase (*p* < 0.05)	100%
Hettinga et al. [27]^a^	45 M and 45 W 5000-m runners and 45 M and 45 W 10,000-m runners in the 2013 and 2017 WC and 2016 OG finals	Official finishing and split times were obtained from IAAF website, published results and from SEIKO	a) In the men’s 5000-m, top-8 athletes were faster than top-15 athletes during the 4100–4200 m and from the 4300–4400 m to the final 100-m segment (*p* < 0.05). Medallists were faster than top-15 athletes from the 4300–4400-m to the final 100-m segment (*p* < 0.05). Medallists were faster than top-8 athletes for the final 100-m segment only (*p* < 0.05) b) In the women’s 5000-m, top-8 athletes were faster than top-15 athletes during the 2700–2800-m and from the 2900–3000-m to the final 100-m segment (*p* < 0.05). Medallists were faster than top-15 athletes from the 2400–2500-m to the final 100-m segment (*p* < 0.05). Medallists were faster than top-8 athletes during the 2600–2700-m, 3000–3100-m, 3800–3900-m to 4400–4500-m and 4600–4700-m to 4800–4900-m segments (*p* < 0.05) c) In the men’s 10,000-m, top-8 athletes were faster than top-15 athletes from 8000–8100-m to 9800–9900-m segment (*p* < 0.05). Medallists were faster than top-15 athletes from 8100–8200-m to the final 100-m segment (*p* < 0.05). Medallists were faster than top-8 athletes from 9600–9700-m to the final 100-m segment (*p* < 0.05) d) In the women’s 10,000-m, top-8 athletes were faster than top-15 athletes during the 1600–1700-m, for all but five of the 100-m segments from 2400–2500-m to 8500–8600-m and for the 9500–9600-m and 9600–9700-m segment (*p* < 0.05). Medallists were faster than top-15 for all but one 100-m segment from 2800–2900-m to the final 100 m (*p* < 0.05). Medallists were faster than top-8 athletes for six 100-m segments during 5000–6000-m, four 100-m segments during 6000–7000-m, two 100-m segments during 7000–8000-m, four 100-m segments during 8000–9000-m and nine 100-m segments during the final 1000 m (*p* < 0.05)	93%
Filipas et al. [29]	73 M and 69 W 5000-m runners and 80 M and 80 W 10,000-m runners in the 2008 and 2016 OG and 2013, 2015 and 2017 WC finals	Race times, 1000-m split times, PB and SB times were obtained from IAAF and the All-Athletics websites	a) In the men’s 5000-m, 1000-m split times were only lower for medallists and top-8 athletes compared with top-16 athletes during the final 1000 m of the race (*p* < 0.001). There was no difference in 1000-m split time between medallists and top-8 athletes b) In the women’s 5000-m, medallists had faster 1000-m split times compared with top-16 athletes during the 3rd split (*p* < 0.05). Medallists and top-8 athletes had faster 1000-m split times compared with top-16 athletes during the 4th and 5th split of the race (*p* < 0.001). Medallists also had faster 1000-m split times compared with top-8 athletes during the final 1000-m of the race (*p* < 0.01) c) In the men’s 10,000-m, medallists and top-8 athletes had faster 1000-m split times during the 8th split (*p* < 0.01) and faster 1000-m split time during the 9th and 10th split compared with top-16 athletes (*p* < 0.001). Medallists had faster 1000-m split times compared with top-8 athletes during the final 1000 m of the race (*p* < 0.001) d) In the women’s 10,000-m, medallists and top-8 athletes had faster 1000-m split times compared with top-16 athletes during the 4th split (*p* < 0.05). Medallists had faster 1000-m split times compared with top-16 athletes during the 5th split (*p* < 0.05). Medallists (*p* < 0.001) and top-8 athletes (*p* < 0.01) had faster 1000-m split times compared with top-16 athletes during the 6th to 9th split. Medallists and top-8 athletes had faster 1000-m split times compared with top-16 athletes during the 10th split (*p* < 0.001). Medallists had faster 1000-m split times compared with top-8 athletes during the 10th split (*p* < 0.001)	93%
*Benchmark performance relative to athlete history*				
Filipas et al. [29]	73 M and 69 W 5000-m runners and 80 M and 80 W 10,000-m runners in the 2008 and 2016 OG and 2013, 2015 and 2017 WC finals	Race times, 1000-m split times, PB and SB times were obtained from IAAF and the All-Athletics websites	a) Better finishing positions were associated with faster PB times across the previous 32 months (*p* < 0.001) for the men’s 5000-m (*r* = 0.575), women’s 5000-m (*r* = 0.800), men’s 10,000-m (*r* = 0.732) and women’s 10,000-m (*r* = 0.611) b) In the men’s 5000-m, medallists and top-8 athletes ran at a lower percentage of their 32-month best time compared with non–top-8 athletes in the last 1000 m (*p* < 0.001, ES = 1.33; *p* = 0.002, ES = 0.99) c) In the women’s 5000-m, medallists completed the first 1000-m split at a higher percentage of 32-month best time compared with non–top-8 athletes (*p* = 0.011, ES = 0.91). Medallists and top-8 athletes ran at a lower percentage of their 32-month best time compared with non–top-8 athletes during the 3000–4000-m (*p* = 0.007, ES = 1.02; *p* = 0.008, ES = 0.87, respectively) and 4000–5000-m split (*p* < 0.001, ES = 1.57; *p* < 0.001, ES = 1.20, respectively) d) In the men’s 10,000-m, medallists and top-8 athletes completed the 2000–3000-m split at a higher percentage of their 32-month best time compared with top-16 athletes (*p* < 0.001, ES = 1.45; *p* = 0.001, ES = 0.86). Conversely, medallists and top-8 athletes completed the 8000–9000-m and 9000–10,000-m splits at a lower percentage of their 32-month best times compared with top-16 athletes (all *p* ≤ 0.001, ES ≥ 0.91) e) In the women’s 10,000-m, medallists completed 1000-m splits from the 7000-m mark onwards at a lower percentage of 32-month best time compared with top-16 athletes (all *p* ≤ 0.037, ES ≥ 0.91). Medallists completed the final 1000-m split at a lower percentage of 32-month best time compared with top-8 athletes (*p* = 0.009, ES ≥ 1.32)	93%

*CT* competition performance time, *ES* effect size, *IAAF* International Association of Athletics Federations, *M* men, *MDR* middle-distance runners, *OG* Olympic Games, *PB* personal best performance time, *SB* season best performance time, *SC* steeplechase, $\dot{V}$*O₂max* maximal oxygen consumption, *W* women, *WC* World Championships

^a^For these studies, only characteristics which showed significant association or capacity to differentiate performance were provided in the overview of main outcome section

**Fig. 5:**
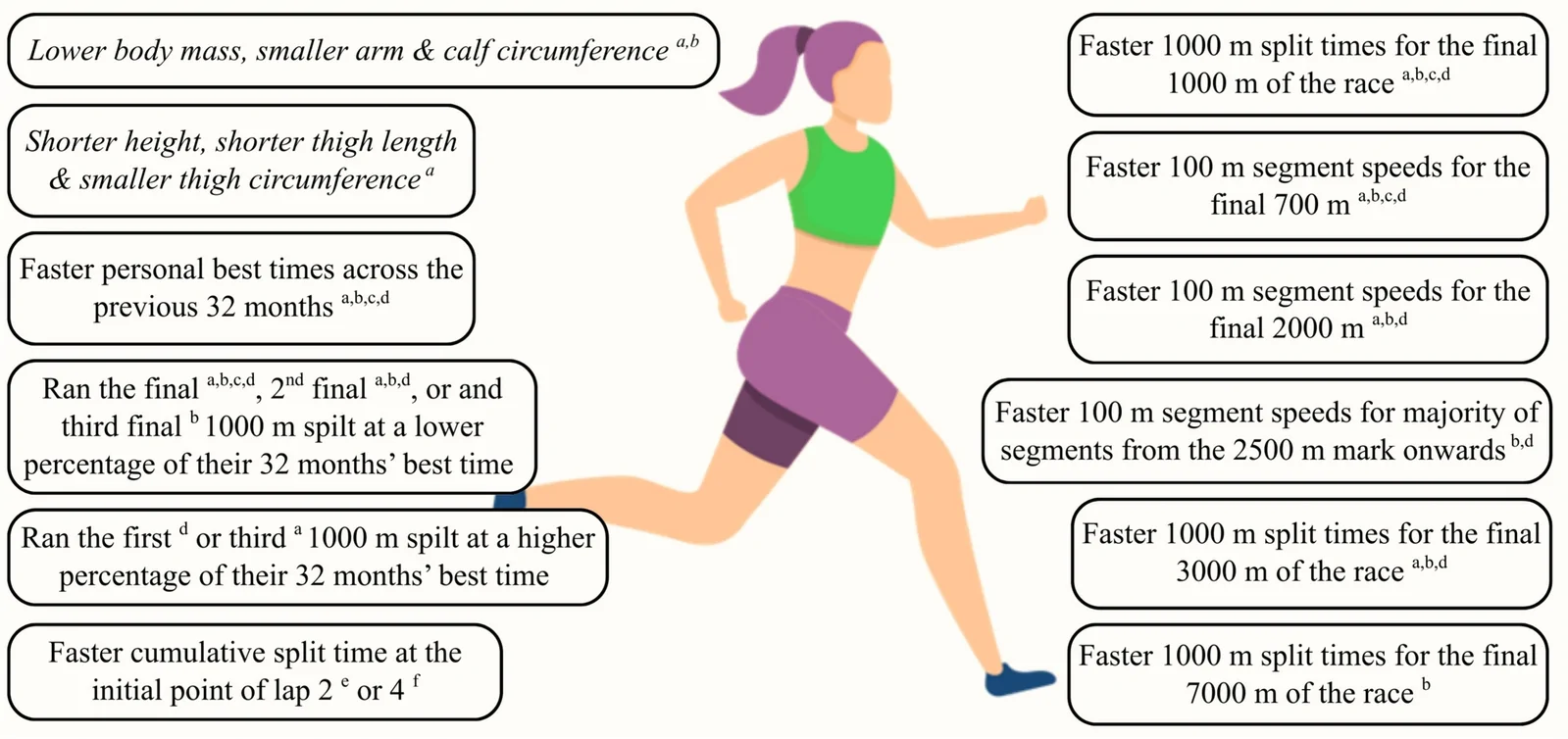
Biological and race strategy characteristics associated with better performance in elite long-distance running. Characteristics differentiated performance among ^a^male 10,000-m runners, ^b^female 10,000-m runners, ^c^male 5000-m runners, ^d^female 5000-m runners, ^e^female 3000-m SC runners, ^f^male 3000-m SC runners. Italics are used to denote only those associations that were significant with the presence of their corresponding covariates. *SC* steeplechase

A summary of biological and race strategy characteristics associated with faster finishing times or better finishing positions among elite LDR is provided in Fig. 5.

##### Anthropometry

Two studies specifically investigated the anthropometric profile of elite 10,000-m runners [76, 79] with one also assessing whether these characteristics differentiated performance within this cohort (Table 5) [76]. Three additional studies reported body mass and height as standard practice for reporting participant demographics [46, 77, 78]. Two further studies reported body surface area [74] and body mass [38] to account for variations in overall body size that may influence other measured characteristics.

In the 3000-m steeplechase, mean height was reported as 1.65 ± 0.05 m for women and 1.78 ± 0.07 m for men [46]. For men, mean body mass was also reported as 62.8 ± 2.7 kg [38]. In elite women’s 10,000-m runners, mean body mass was 47.3 ± 3.5 kg and height was 1.63 ± 0.03 m [76]. Reported limb characteristics included mean thigh length of 37.9 ± 1.6 cm, arm length of 24.5 ± 1.2 cm, forearm length of 26.3 ± 2.6 cm, arm circumference of 22.9 ± 3.6 cm, thigh circumference of 36.9 ± 4.1 cm and calf circumference of 23.3 ± 6.5 cm [76]. In elite men’s 10,000-m runners, weighted mean body mass was 58.6 kg and height was 1.74 m [76, 77, 79]. Weighted mean thigh length, thigh circumference and calf circumference were 41.4 cm, 45.6 cm and 32.1 cm, respectively [76, 79]. Additionally, mean arm length was 29.0 ± 3.5 cm, forearm length was 27.6 ± 4.3 cm and arm circumference was 29 ± 3.5 cm [76]. Relative segment proportions were also reported, included a leg to height ratio of 0.48 ± 0.01, shank to height ratio of 0.22 ± 0.01 and shank to thigh ratio of 1.08 ± 0.05 [79].

##### Physiology

Physiological characteristics were reported for male 3000-m steeplechase runners [38] and for male 10,000-m runners [11, 77, 79]. Two of these studies found no associations between physiological characteristics and performance among elite LDR [38, 79] (Table 5).

For elite 10,000-m runners, Santos-Concejero et al. [79] reported a mean running economy of 191.4 mL·kg⁻^1^·km⁻^1^ at 19 km·h⁻^1^, whereas Yamanaka et al. [11] reported a running economy of approximately 58 mL·kg⁻^1^·min⁻^1^ at 18 km·h⁻^1^. Elite steeplechasers demonstrated a mean relative $\dot{V}$O₂max of 70.4 ± 1.9 mL·kg⁻^1^·min⁻^1^ [38], while a weighted mean relative $\dot{V}$O₂max of 77.29 mL·kg⁻^1^·min⁻^1^ was reported for elite 10,000-m runners [77, 79]. Maximal heart rate was reported as a mean of 190 ± 10 bpm for elite male steeplechasers [38] and 180 ± 4 bpm for elite male 10,000-m runners [77]. Additionally, the energy cost of running at 70% of $\dot{V}$O₂max was reported as 3.96 ± 3.960.32 J.kg^−1^.m^−1^ for elite steeplechasers [38].

##### Biomechanics

Biomechanical characteristics of elite 3000-m steeplechasers were reported based on competition video analysis [46, 83]. Hanley et al. [46] analysed 3000-m steeplechase runners as they jumped over the last water jump in the 2017 World Championship final. Elite male steeplechase runners had an approach step length of 1.72 ± 0.12 m, take-off distance of 1.53 ± 0.15 m, clearance height of 0.67 ± 0.06 m, landing distance of 2.71 ± 0.35 m and exit step length of 1.15 ± 0.07 m [46]. Elite female steeplechase runners had an approach step length of 1.60 ± 0.16 m, take-off distance of 1.37 ± 0.18 m, clearance height of 0.64 ± 0.04 m, landing distance of 2.06 ± 0.49 m and exit step length of 1.04 ± 0.14 m [46]. Further, better water jump clearances were associated (*p* < 0.05) with greater landing distances, approach velocities and exit velocities for both sexes [46]. Chortiatinos et al. assessed hurdle clearance mechanics in an elite female steeplechase runner [83].

Biomechanics of elite male 10,000-m runners have been investigated using both competition video analysis of championship races [78, 80, 81] and an optical measurement system (OptoJump) during a treadmill speed test designed to replicate race pace [79]. Three studies reported step length and step frequency [78–80], which remained relatively consistent across studies, with mean values of 1.95 ± 0.12 m [80], mean of approximately 1.90 m [79] and values ranging between 1.80 m and 2.05 m [78]. Greater variation between studies existed for step frequency, with mean values of 3.09 ± 0.18 Hz [80], mean of approximately 3.25 Hz [79] and values ranging between 3.1 Hz and 3.6 Hz [78]. Two studies also reported contact times, with similar values observed across studies, approximately 0.18 s [79] and 0.17–0.18 s [80]. Hanley et al. [80] additionally reported joint angles at toe-off and initial contact, including a knee angle at toe-off of 159 ± 5°, ankle angle at toe-off of 126 ± 3°, elbow angle at toe-off of 49 ± 7°, hip angle at contact of 149 ± 3°, knee angle at contact of 150 ± 5° and ankle angle at contact of 108 ± 6°. Finally, elite male 10,000-m runners were either midfoot or forefoot strikers and were able to alternate between these anterior footstrike patterns during the race [81]. There was no difference in the number of runners who were midfoot or forefoot strikers at any of the analysis points during the race [81].

Biomechanical characteristics of elite female 10,000-m runners were reported based on competition video analysis [82]. Hanley et al. [82] reported spatiotemporal characteristics including a mean step length of 1.79 ± 0.07 m, step frequency of 3.09 ± 0.10 Hz and contact time ranging between 0.16 s and 0.17 s. Joint angles at toe-off and initial contact were also reported, including a mean knee angle at toe-off of 166 ± 4°, ankle angle at toe-off of 131 ± 5°, elbow angle at toe-off of 55 ± 12°, hip angle at contact of 149 ± 4°, knee angle at contact of 153 ± 3° and ankle angle at contact of 112 ± 4° [82].

##### Morphology

A longitudinal study assessed heart morphology of an elite male and female 5000-m runner [59]. Increases in LVIDd (*p* = 0.019) and decreases in PWT (*p* < 0.05) were associated with performance improvements for the elite 5000-m male runner but not the female runner [59]. Another study reported heart morphological characteristics, including LVIDd and PWT, at a single timepoint [74].

##### Pacing

Seven studies reported pacing characteristics from a single benchmark event [30, 78, 80–82, 84, 85], whereas three studies analysed multiple benchmark events [27, 29, 86]. Further, three studies [27, 29, 86] investigated whether pacing characteristics differentiated performance (Table 5).

In the 3000-m steeplechase, lap segment speed revealed a U-shaped pacing profile for top-8 athletes in the men’s race and for both top-8 and non–top-8 athletes in the women’s race [86]. This pacing pattern involved a relatively fast start, a slower middle segment and a finishing surge [86]. Non–top-8 athletes in the men’s race followed a similar pacing profile; however, they did not increase their speed in the final stage of the race and instead maintained their pace [86]. Pace variability, calculated using the coefficient of variation between and within laps, ranged from 2.4 to 3.2% among elite 3000-m steeplechasers [86]. Top-8 athletes demonstrated slightly lower lap-to-lap and within-lap variability compared with non–top-8 athletes [86].

In general, pacing profiles of the 5000-m and 10,000-m races showed slightly varying pace until the end of the race, where athletes increased their speed [27]. Pace variability based on the coefficient of variation of all 100-m segment speeds was on average 3.6–11.4% [27, 30], with greater pace variability reported for medallists compared with top-8 athletes [27]. In the men’s and women’s 5000-m and 10,000-m races, medallists adopted a negative pacing profile, running faster in the second half of the race [27, 30]. In most cases, top-8 and top-15/16 athletes also adopted a negative pacing profile, but this pacing profile was closer to even compared with medallists and was positive in some instances [27, 30].

##### Tactics

Three studies reported tactical characteristics from a race at a benchmark event [60, 84, 85].

##### Qualification Patterns

One study reported qualification patterns at 16 benchmark events [68]. Qualifying times in the heats were slower (*p* < 0.001) than SB times for men’s medallists (*d* = 2.74), women’s medallists (*d* = 1.99), men’s top-8 non-medallists (*d* = 2.40) and women’s top-8 non-medallists (*d* = 1.12) in the 5000-m [68].

##### Benchmark Performance Relative to Athlete History

Five studies reported benchmark performance relative to athlete history for one benchmark event [80–82, 84, 85] and two studies analysed multiple benchmark events [29, 68]. Of these studies, one examined whether benchmark performance relative to athlete history differentiated performance among elite 5000-m and 10,000-m runners (Table 5) [29].

Finishing time relative to 32-month best time showed that all finalists, except medallists in the women’s 10,000-m, ran times slower than their 32-month best time [29]. Finishing times in the final were slower than SB times for men’s medallists (*p* < 0.001; *d* = 1.32), women’s medallists (*p* < 0.001; *d* = 0.77), men’s top-8 athletes (*p* < 0.001; *d* = 1.43) and women’s top-8 athletes (*p* < 0.01; *d* = 0.4) in the 5000-m [68].

#### Discussion for Long-Distance Running

##### Anthropometry

Anthropometric characteristics were largely reported for descriptive purposes or to account for variations in overall body size that could affect other assessed outcomes [38, 46, 74, 77, 78], with only two studies specifically investigating the anthropometric profile of elite LDR [76, 79]. Anthropometric characteristics were inconsistently reported across events and sexes, with more detailed profiling in the 10,000-m compared with minimal or absent reporting in the 5000-m. Consequently, understanding of anthropometric characteristics in elite LDR is largely based on evidence from 10,000-m runners. Although current evidence provides an indication for coaches and athletes of typical anthropometric characteristics for elite 10,000-m runners, it is important to acknowledge that at least 41 of the 49 elite athletes were of East African origin [76–79]. Whilst this reflects the dominance of East African runners in long-distance events [76, 87], these findings may not be generalisable to the broader population of elite LDR.

One of these studies specifically assessed the capacity of anthropometric characteristics to differentiate performance among elite 10,000-m runners [76]. Several characteristics, including lower body mass and smaller limb circumferences, were associated with faster finishing times among elite 10,000-m runners [76]. These findings reflect the potential advantage of smaller body dimensions in long-distance running [76], as reduced body mass and limb circumference may decrease the muscular effort required for limb acceleration and thereby reduce the metabolic cost of running [21, 88]. However, none of these anthropometric characteristics showed independent associations with performance when controlling for covariates, suggesting they may not independently determine performance within elite 10,000-m runners [76]. Consequently, performance differences may relate more to the overall anthropometrical profile of the elite LDR rather than any single characteristic. From a practical perspective, anthropometric profiling may therefore provide useful contextual information for athlete development, on the condition that athlete health and wellbeing are prioritised.

Anthropometric characteristics have been compared between elite Eritreans and near-elite Europeans [79]. Consistent with findings in elite 10,000-m runners [76], elite Eritrean runners demonstrated smaller calf circumference (*p* < 0.01) than near-elite European runners [79]. The consistently smaller calf circumference observed among elite runners may suggest a performance determinant of elite long-distance running. However, the comparison between East African elite and European near-elite runners also highlights the potential influence of population-specific anthropometric characteristics. No other anthropometric characteristics including body mass, height, thigh length and circumference, differed between these populations [79], suggesting that these individual characteristics may have limited capacity to differentiate performance among high level runners.

Evidence investigating highly trained but non-elite runners further highlights the variability in associations between anthropometry and performance across performance standards. Mooses et al. [89] reported that longer legs were associated with better running performance, while no associations were observed for body mass or limb circumference. These findings [89] contrast with observations in elite 10,000-m runners [76] and between elite and near-elite runners [79], suggesting that anthropometric determinants of performance differ according to performance standard.

##### Physiology

Current evidence provides some insight into the physiology of elite male 3000-m steeplechasers and 10,000-m runners [11, 38, 77, 79]. The reported values for maximal heart rate, $\dot{V}$O₂max, energy cost of running and running economy provide an indication for coaches and athletes of typical physiological characteristics among elite LDR. However, these values are derived from studies of male 3000-m steeplechasers and 10,000-m runners and are based on relatively small sample sizes, limiting generalisability. Additionally, current evidence does not provide insight into the physiology of elite female LDR. Further, findings suggest that physiological characteristics including running economy and $\dot{V}$O₂max may not be capable of differentiating performance among elite LDR [38, 79].

Santos-Concejero et al. [79] found that average running economy was not associated with 10,000-m performance among elite male Eritrean runners. However, when elite Eritrean and near-elite European runners were analysed together, average running economy was associated (*r* =  − 0.57; *p* = 0.013) with 10,000-m performance [79]. These findings suggest that running economy may be more capable of differentiating performance among athletes of more varied performance. Similarly, among athletes of lower performance standards, running economy was associated with 5000-m performance in highly trained male runners but not with 10,000-m performance in near-elite runners [11]. Collectively, these findings indicate that while running economy may differentiate athletes across broader ranges of performance, its ability to differentiate performance among elite LDR appears unlikely. Further research is required to determine whether running economy can differentiate performance within elite cohorts. Additionally, elite Eritrean runners demonstrated better running economy than near-elite European runners at 19 km·h⁻^1^, but not at 17 or 21 km·h⁻^1^ [79], highlighting the potential influence of assessment speed when evaluating running economy.

Tartaruga et al. [38] found no correlation between absolute, relative or allometrically scaled $\dot{V}$O₂max and performance among elite steeplechasers. Similarly, studies including highly trained [15, 37] and near-elite [11, 22] endurance runners have reported no association between $\dot{V}$O₂max and performance. Collectively, these findings suggest that although $\dot{V}$O₂max is important for long-distance running, it may have limited ability to differentiate performance among high-performing LDR. Supporting this interpretation, relative $\dot{V}$O₂max values reported for elite 3000-m steeplechasers [38] were slightly lower than those observed in near-elite steeplechasers [22, 90, 91], indicating that athletes of lower performance standards may possess similar or even higher $\dot{V}$O₂max values. Similarly, comparable relative $\dot{V}$O₂max values have been reported for near-elite male 10,000-m runners [91–93] and elite 10,000-m runners [77, 79]. These findings reinforce that $\dot{V}$O₂max may not differentiate performance among high-performing LDR. In contrast, Zhou et al. [77] reported higher (*p* < 0.001) relative $\dot{V}$O₂max values in elite 10,000-m runners compared with trained runners and students [77]. These findings suggest that $\dot{V}$O₂max may be capable of differentiating performance among athletes of significantly different performance standards (e.g. elite compared with trained runners) but not between athletes closer in performance standards (e.g. elite compared to near-elite runners).

Findings among trained, highly trained and near-elite LDR revealed associations between better performance and faster 100-m and 400-m sprint times [11], greater velocity at $\dot{V}$O₂max [13, 17, 72] and greater velocity at 4 mmol·L blood lactate [13, 72]. Based on this evidence [11, 13, 17, 72], there is good theoretical grounding for future research to investigate whether these characteristics can differentiate performance among elite LDR. However, as observed for $\dot{V}$O₂max and running economy, associations between these physiological characteristics and performance may not persist among elite cohorts.

#### Biomechanics

Current evidence provides some insight into the biomechanics of elite 3000-m steeplechasers and 10,000-m runners [46, 78–83]; however, no studies have specifically examined these characteristics in elite 5000-m runners. The different barrier tasks examined across studies investigating elite 3000-m steeplechasers limit the direct comparison of findings [46, 83]. Additionally, having only a single athlete investigated in the study by Chortiatinos et al. [83] restricts the extent to which these findings can be generalised to the broader elite long-distance running population. Hanley et al. [46] found that faster water jump clearances were associated with greater landing distance, approach velocity and exit velocity. These findings suggest that steeplechasers benefit from approaching the barrier at high speed and generating a strong push off to achieve a greater landing distance to help reduce time lost when exiting the water. The values reported for elite 3000-m steeplechasers provide a useful reference for coaches and athletes regarding typical water jump mechanics at benchmark events [46]. However, considerable variation in several characteristics was observed, indicating that elite athletes may employ a range of technical approaches when negotiating the water jump [46].

The spatiotemporal, footstrike pattern type and joint angle values reported for elite 10,000-m runners provide an indication of typical running mechanics at race pace for coaches and athletes [78–81]. Across studies, step length and contact time appeared relatively consistent despite different data collection methods [78–80], suggesting that elite 10,000-m runners may adopt relatively stable step lengths and contact times when running at race pace. In contrast, greater variation was observed for step frequency [78–80], suggesting that this characteristic may be more responsive to changes in running conditions. Elite runners adopted both midfoot and forefoot strike patterns and were able to transition between these patterns [81], highlighting the importance of running with an anterior striking pattern for elite 10,000-m performance. Further, the absence of differences in the prevalence of these footstrike patterns across race segments [81] indicates that footstrike type may not differentiate performance among elite LDR.

Evidence examining the biomechanics of elite female 10,000-m runners is limited, with only one study reporting spatiotemporal and joint angle characteristics [82]. While these findings provide an initial indication of typical running mechanics in elite female LDR [82], the limited evidence makes it difficult to determine whether these characteristics are consistent across competitions.

No studies investigated whether biomechanics could differentiate performance among elite LDR. Santos-Concejero et al. [79] compared elite Eritrean to near-elite European runners and found no difference between groups for contact time, swing time, step length or frequency at three assessed speeds. Consequently, these spatiotemporal characteristics may not be capable of differentiating performance among high performing LDR. However, future research should assess whether other biomechanical characteristics are associated with performance among elite LDR.

##### Morphology

A longitudinal study assessed heart morphology of an elite male and female 5000-m runner [59]. Increases in LVIDd (*p* = 0.019) and decreases in PWT (*p* < 0.05) were associated with performance improvements for the elite 5000-m male runner but not the female runner [59]. Another study reported heart morphological characteristics, including LVIDd and PWT, for two elite female 5000-m runners [74].

Evidence investigating heart morphology in elite LDR is limited [59, 74]. Longitudinal investigation into heart morphology found that increases in LVIDd and decreases in PWT were associated with improvements in individual performance in one elite long-distance runner but not the other [59]. Further, evidence from near-elite runners provides limited contextual insight. Greater LVIDd was associated with faster 10,000-m performance among near-elite male runners, although no associations were observed in other events [74]. However, these findings should be interpreted cautiously due to the small sample sizes in several groups [74]. Current evidence is insufficient to understand whether heart morphological characteristics differentiate performance among elite LDR; further research in this cohort is warranted.

##### Pacing

Current evidence provides insight into pacing strategies used by elite LDR at benchmark events [27, 29, 30, 78, 80–82, 84–86]. Specifically, Hanley and Williams [86] provides some insight into pacing characteristics of elite 3000-m steeplechase runners by investigating the men’s and women’s final at two benchmark events. Top-8 athletes demonstrated lower cumulative split times earlier in the race compared with non–top-8 athletes in both the men’s and women’s 3000-m steeplechase [86]. These findings may indicate that more successful athletes adopt a faster early pacing strategy, which may reduce the need for large increases in pace later in the race. However, pacing profiles revealed that more successful 3000-m steeplechase athletes were better able to increase speed during the closing stages of the race [86]. Additionally, the slightly lower pacing variability observed in top-8 athletes [86] may indicate that the ability to maintain a more consistent pace throughout the race is advantageous. Further, pacing variability was relatively low for all athletes [86]; the ability to sustain pace may be more important for performance than overall variability in lap pace.

Hettinga et al. [27] and Filipas et al. [29] provided insight into whether pacing characteristics differentiate performance among elite 5000-m and 10,000-m runners by investigating the men’s and women’s finals at five benchmark events. In the women’s races, differences in 100-m segment speed between top-15 athletes and medallists or top-8 athletes emerged from halfway in the 5000-m and earlier during the 10,000-m [27]. Similarly, differences in 1000-m split time between top-16 athletes and medallists or top-8 athletes emerged from the 2000–3000-m split onwards in the 5000-m, and 3000–4000-m split onwards in the 10,000-m [29]. This highlights that in women’s 5000-m and 10,000-m races, it is important to be able to keep up with the lead group, especially after the halfway mark, to achieve a top-8 position [27, 29]. Further, in women’s races, medallists ran 100-m segments faster than top-8 athletes primarily during a majority of the final 1000 m [27] and medallists compared to top-8 athletes had faster 1000-m split times for the final 1000 m [29]. Being able to respond to increases in speed, especially in the final 1000 m, seems important for podium success in women’s long-distance races.

In contrast, differences in the men’s races between top-15 athletes and medallists or top-8 athletes occurred later, predominantly during the final two laps of the 5000-m and the last 2000 m of the 1000-m [27]. Similarly, differences in 1000-m split time between top-16 athletes and medallists or top-8 athletes emerged in the final 1000 m of the 5000-m and from the 7000–8000-m split onwards in the 10,000-m [29]. This indicates that finalists in men’s 5000-m and 10,000-m races are more evenly matched, with pacing characteristics differentiating athletes only in the final stages of the race [27, 29]. Further, in the men’s race, medallists were faster than top-8 athletes only in the final 100 m of the 5000-m and the last lap of the 10,000-m [27]. Medallists compared with top-8 athletes only had faster 1000-m split times for the final 1000 m of the men’s 10,000-m [29]. These findings suggest that the ability to increase pace during the closing stages of the race may be important for securing a medal, highlighting the importance of strong finishing speed in men’s long-distance benchmark events.

Hettinga et al. [27] and Thiel et al. [30] provide an understanding of pace variability and pacing differentials in the 5000-m and 10,000-m based on four benchmark events. More successful athletes appear better able to increase pace during the second half of the race, indicating that the capacity to respond to late race demands may be an important determinant of performance success [27, 30]. The slightly greater pacing variability observed among medallists [27] may therefore reflect a greater ability to respond to tactical changes. In contrast, athletes finishing outside medal positions appeared to maintain pacing strategies that were closer to even [27, 30], which may indicate a reduced capacity to respond to accelerations during critical late stages of the race. Collectively, these findings suggest that the ability to tolerate and respond to pace changes, particularly in the latter stages of championship races, may be important for success in elite long-distance running.

##### Tactics

Current evidence provides limited insight into tactical strategies used by elite LDR at benchmark events, with three low-quality studies investigating a single race [60, 84, 85]. Consequently, it is difficult to draw conclusions regarding tactical characteristics in elite long-distance running.

No studies have investigated whether tactical characteristics can differentiate performance among elite LDR. However, research examining long-distance races across benchmark and non-benchmark events has identified key tactical characteristics of male gold medallists in the 5000-m, including maintaining a position within the top five throughout the race and being in the top three at the initiation of final sprint [94]. This highlights the potential importance of race positioning and tactical decision making in determining race outcomes. Further research investigating tactics in elite long-distance running races is needed.

##### Qualification Patterns

Current evidence provides an insight into qualification times for elite 5000-m runners participating in 16 benchmark events [68]. Qualifying times in the heats were slower than SB time for medallists and non-medallists [68], highlighting the importance of conserving energy during the qualification race. Whilst the capacity of qualification patterns to differentiate performance was not investigated, they are likely less relevant in long-distance events which involve a single qualification round and include more finalists than middle-distance events.

##### Benchmark Performance Relative to Athlete History

Current evidence provides some insight into benchmark performance relative to athlete history for elite 5000-m and 10,000-m runners [29, 68, 80–82, 84, 85], specifically as two of these studies analysed multiple benchmark events [29, 68]. Further, one study investigated whether performance relative to an athlete’s historical best differentiates success [29].

Faster historical 32-month best time and the ability to utilise this capacity during key stages of the race differentiated performance in the 5000-m and 10,000-m at benchmark events [29]. Faster best time for the previous 32 months showed large to very large correlations with better finishing position across all events [29], indicating that historical performance capacity is an important determinant of benchmark event success. Medallists and top-8 athletes ran closer to their 32-month best time in the final 1000-m split of the 5000-m and the final 2000-m split of the 10,000-m compared with top 15/16 athletes, in all cases except the women’s 10,000-m, in which only medallists did [29]. This pattern suggests that being able to run closer to one’s historical best time in the final moments of the race may be crucial for separating more successful from less successful athletes at benchmark events. Differences in the early stages of races were less consistent; however, medallists in the women’s 5000-m and medallists and top-8 athletes in the men’s 10,000-m completed the 1st and 3rd 1000-m splits, respectively, at a pace slower than their 32-month best time when compared with top 15/16 athletes [29]. As better-placed finishers generally had faster historical best times, they were able to run at a similar absolute pace while running further below their historical best times, potentially allowing these athletes to conserve more energy for the final moments of the race.

Finishing time relative to athletes’ 32-month best time showed that all finalists, except medallists in the women’s 10,000-m, ran times slower than their 32-month best time [29]. Similarly, medallists and non-medallists were slower than their SB times in the final [68]. These findings highlight that championship races are rarely run at maximal speeds and that tactical strategies play an important role in determining success. While having a fast historical best time is clearly important for elite LDR, success at benchmark events may also depend on being physically prepared to respond to tactical changes in pace.

### Limitations

Some limitations should be considered when interpreting the findings of this systematic review. Studies were only included if participants’ performance times met the inclusion standard or if participants were top-16 finalists from benchmark events. Many studies, however, referred to participants as ‘elite’ or ‘international’ without providing performance times and were therefore excluded from the review. Whilst authors of relevant studies published prior to 2000 were contacted, few responded, potentially resulting in the exclusion of studies that met the inclusion criteria but lacked sufficient available information. Additionally, comparing findings between studies investigating biological characteristics was difficult due to variability in data collection and analysis methods used. For example, studies including biomechanical characteristics often collected data using different equipment at different sampling rates and analysed data using different software and models, rendering data comparison difficult. Few studies were available investigating the capacity of characteristics to differentiate performance among elite endurance track runners, resulting in athlete profiles rather than conclusive evidence of characteristics that define performance success at benchmark events. Data extraction was completed by one reviewer, which increases the risk of errors and subjective bias. Several studies investigating race strategies analysed the same benchmark events. When identical characteristics were examined across studies, duplicate findings were removed; however, studies analysing different race strategy characteristics were retained. For example, both Filipas et al. [29] and Hettinga et al. [27] analysed the men’s and women’s 5000-m and 10,000-m finals at the 2013 and 2017 World Championships and the 2016 Olympic Games. However, Filipas et al. [29] reported 1000-m split times, whereas Hettinga et al. [27] reported 100-m segment speeds, and therefore both findings were included in this review. Consequently, similar trends may reflect the use of the same underlying race data, which may overstate the strength of these findings. A substantial proportion of included articles were from World Athletics publications, as relevant evidence in this area is not fully captured by standard academic databases. Performance was defined as either finishing time or position at benchmark events or recent PB time. These differing criteria could have led to different findings between studies. However, this approach was necessary to allow analysis of all characteristics relative to performance. Only studies conducting competition analysis could assess performance relative to finishing time or position at benchmark events.

## Conclusion

This review provides a good understanding of current evidence investigating biological and race strategy characteristics among endurance track runners capable of competing at benchmark events. These findings can be used to develop profiles on what is required to be an elite endurance runner, helping to aid in the identification and development of athletes within high-performance athletics programmes. However, due to the limited current evidence investigating biological characteristics and the often small sample sizes included in these studies, it is difficult to understand the optimal biological profile of this population. Race strategy characteristics provide a greater understanding of elite endurance track running. Characteristics previously shown to be capable of differentiating performance among elite endurance track runners have also been identified, highlighting potential key performance differentiators. High-performance athletics programmes can use these findings to help guide talent identification and athlete development. An important ‘takeaway’ is that pacing characteristics associated with speed or time over the final segments of the race were consistently able to differentiate performance in elite endurance track running events. All other characteristics found to differentiate performance were only investigated in one study, and future research should investigate these characteristics further to determine whether they can consistently differentiate performance among elite endurance track runners. Additionally, limited evidence investigated the capacity of characteristics, especially in the biological domain, to differentiate performance. Further research is needed to gain a more holistic understanding of characteristics capable of differentiating performance among elite endurance track runners.

## Supplementary Information

Below is the link to the electronic supplementary material.

Supplementary file1 (DOCX 73 KB)

## Declarations

### Availability of data and material

Data relevant to the study are included in the article or available on request from the corresponding author.
