# Supplementary material for: Biological and Race Strategy Characteristics Investigated in Elite Endurance Track Running: A Systematic Review
Source: Sports Med. 2026 Jul 9;56(11):2733–65. doi: 10.1007/s40279-026-02475-8 (PMC13672677; doi:10.1007/s40279-026-02475-8)
Supplement: Supplementary file 1 [file 40279_2026_2475_MOESM1_ESM.docx]

Supplementary information

Supplementary Table 1. Sarmento et al. 2017 checklist [49] results for all included studies

| Study | Items | | | | | | | | | | | | | | | Total (%) | Rating |
| --- | --- | --- | --- | --- | --- | --- | --- | --- | --- | --- | --- | --- | --- | --- | --- | --- | --- |
|  | 1 | 2 | 3 | 4 | 5 | 6 | 7 | 8 | 9 | 10 | 11 | 12 | 13 | 14 | 15 |  |  |
| Carter et al. 1967 [53] | 0 | 1 | N/A | 1 | 1 | 1 | 0 | 1 | 0 | N/A | 1 | 1 | 1 | 0 | 0 | 62 | G |
| Casado et al. 2020 [68] | 1 | 1 | 1 | 1 | 0 | N/A | 1 | 1 | 1 | 1 | 1 | 1 | 1 | 1 | 0 | 86 | E |
| Chortiatinos et al. 2010 [83] | 1 | 1 | 1 | 1 | 0 | 0 | 0.5 | 0.5 | 1 | 0 | 0 | 1 | 1 | 1 | 0 | 60 | G |
| Dessalew et al. 2019 [76] | 1 | 1 | 1 | 1 | 1 | 1 | 1 | 1 | 1 | 1 | 1 | 1 | 1 | 1 | 0 | 93 | E |
| Enomoto et al. 2008 [78] | 1 | 1 | 1 | 1 | 0 | 0 | 0 | 0 | 0 | 0 | 0 | 1 | 1 | 1 | 0 | 47 | L |
| Filipas, Torre et al. 2018 [29] | 1 | 1 | 1 | 1 | 1 | N/A | 1 | 1 | 1 | 1 | 1 | 1 | 1 | 1 | 0 | 93 | E |
| González-Mohíno et al. 2021 [28] | 1 | 1 | 1 | 1 | 0 | N/A | 1 | 0.5 | 1 | 1 | 1 | 1 | 1 | 1 | 0 | 82 | E |
| Hanley & Bissas 2018 [67] | 0 | 0 | N/A | 1 | 0 | N/A | 1 | 1 | 0 | 0 | 0 | 0 | 1 | 0 | 0 | 31 | L |
| Hanley & Bissas 2018 [61] | 0 | 0 | N/A | 1 | 0 | N/A | 1 | 1 | 0 | 0 | 0 | 0 | 1 | 0 | 0 | 31 | L |
| Hanley & Bissas 2018 [66] | 0 | 0 | N/A | 1 | 0 | N/A | 1 | 1 | 0 | 0 | 0 | 0 | 1 | 0 | 0 | 31 | L |
| Hanley & Bissas 2018 [62] | 0 | 0 | N/A | 1 | 0 | N/A | 1 | 1 | 0 | 0 | 0 | 0 | 1 | 0 | 0 | 31 | L |
| Hanley & Bissas 2018 [85] | 0 | 0 | N/A | 1 | 0 | N/A | 1 | 1 | 0 | 0 | 0 | 0 | 1 | 0 | 0 | 31 | L |
| Hanley & Bissas 2018 [84] | 0 | 0 | N/A | 1 | 0 | N/A | 1 | 1 | 0 | 0 | 0 | 0 | 1 | 0 | 0 | 31 | L |
| Hanley & Hettinga 2018 [69] | 1 | 1 | 1 | 1 | 0 | N/A | 1 | 1 | 1 | 1 | 1 | 1 | 1 | 1 | 0 | 86 | E |
| Hanley & Williams 2020 [86] | 1 | 1 | 1 | 1 | 1 | N/A | 1 | 1 | 1 | 1 | 1 | 1 | 1 | 1 | 1 | 100 | E |
| Hanley et al. 2018 [80] | 0 | 0 | N/A | 1 | 0 | N/A | 1 | 1 | 1 | 0 | 0 | 0 | 0 | 1 | 0 | 38 | L |
| Hanley et al. 2018 [82] | 0 | 0 | N/A | 1 | 0 | N/A | 1 | 1 | 1 | 0 | 0 | 0 | 0 | 1 | 0 | 38 | L |
| Hanley et al. 2019 [64] | 1 | 1 | 1 | 1 | 0 | N/A | 1 | 1 | 1 | 1 | 1 | 1 | 1 | 1 | 0 | 86 | E |
| Hanley et al. 2020 [46] | 1 | 1 | 1 | 1 | 1 | N/A | 0.5 | 1 | 1 | 1 | 1 | 1 | 1 | 1 | 1 | 96 | E |
| Hanley et al. 2021 [81] | 1 | 1 | 1 | 1 | 1 | 1 | 1 | 0.5 | 1 | 1 | 1 | 1 | 1 | 1 | 1 | 97 | E |
| Hanley et al. 2022 [57] | 1 | 1 | 1 | 1 | 0 | 1 | 0.5 | 0.5 | 1 | 1 | 1 | 1 | 1 | 1 | 1 | 87 | E |
| Hanley et al. 2023 [55] | 1 | 1 | 1 | 1 | 0 | N/A | 1 | 0.5 | 1 | 1 | 0.5 | 1 | 1 | 1 | 1 | 86 | E |
| Hettinga et al. 2019 [27] | 1 | 1 | 1 | 0 | 1 | N/A | 1 | 1 | 1 | 1 | 1 | 1 | 1 | 1 | 1 | 93 | E |
| Ingham et al. 2012 [54] | 0 | 0 | N/A | 1 | 0 | 0 | 1 | 1 | 1 | 1 | 0 | 1 | 1 | 1 | 0 | 57 | G |
| Jones & Whipp 2002 [60] | 0 | 0 | N/A | 1 | 0 | N/A | 0 | 0 | 0 | N/A | 0 | 1 | 1 | 1 | 0 | 33 | L |
| Lacour et al. 1990 [16] | 1 | 1 | 1 | 1 | 0 | 0 | 1 | 0.5 | 1 | 1 | 0 | 0 | 0 | 1 | 0 | 57 | G |
| Legaz, González & Lacambra 2005 [59] | 1 | 1 | 1 | 0 | 1 | 0 | 1 | 1 | 0 | 1 | 1 | 1 | 0 | 1 | 1 | 73 | G |
| Legaz, Ostáriz et al. 2005 [74] | 1 | 1 | 1 | 1 | 0 | 0 | 1 | 1 | 1 | 1 | 1 | 0 | 0 | 1 | 0 | 67 | G |
| Leskinen et al. 2009 [52] | 1 | 1 | 1 | 1 | 0 | 0 | 0.5 | 0.5 | 1 | 1 | 1 | 1 | 1 | 1 | 0 | 73 | G |
| Sandford et al. 2018 [65] | 1 | 1 | 1 | 0 | 0 | N/A | 1 | 1 | 1 | 0 | 1 | 1 | 0.5 | 1 | 0 | 68 | G |
| Sandford et al. 2019 [25] | 1 | 1 | 1 | 1 | 0 | 1 | 1 | 0.5 | 1 | 0 | 1 | 1 | 1 | 1 | 1 | 83 | E |
| Sandford et al. 2019 [63] | 1 | 1 | 1 | 0 | 0 | N/A | 1 | 0.5 | 1 | 0 | 1 | 1 | 1 | 1 | 1 | 75 | G |
| Santos-Concejero et al. 2015 [79] | 1 | 1 | 1 | 1 | 0 | 1 | 1 | 0.5 | 1 | 1 | 1 | 1 | 0.5 | 1 | 1 | 87 | E |
| Skof & Stuhec 2004 [58] | 1 | 0 | 1 | 1 | 0 | 0 | 0.5 | 0.5 | 0 | N/A | 0 | 0 | 0.5 | 0 | 0 | 32 | L |
| Stellingwerff 2018 [56] | 1 | 0 | 1 | 1 | 0 | 1 | 1 | 1 | 0 | 1 | 1 | 1 | 1 | 1 | 0 | 73 | G |
| Tartaruga et al. 2014 [38] | 1 | 1 | 1 | 1 | 1 | 1 | 1 | 1 | 1 | 1 | 0 | 1 | 1 | 1 | 0 | 87 | E |
| Thiel et al. 2012 [30] | 1 | 1 | 1 | 1 | 0 | N/A | 1 | 1 | 1 | 0 | 1 | 1 | 1 | 1 | 0 | 79 | E |
| Yamanaka et al. 2020 [11] | 1 | 1 | 1 | 1 | 0 | 1 | 1 | 0.5 | 0 | N/A | 1 | 1 | 1 | 1 | 0 | 75 | G |
| Zhou et al. 2001 [77] | 1 | 1 | 1 | 1 | 0 | 1 | 1 | 1 | 1 | 1 | 1 | 1 | 1 | 0 | 0 | 80 | E |
| 0= item not addressed, 0.5 = item partial addressed, 1 = item addressed, N/A= item not applicable to study. Rating: L=low, G= good, E= excellent. | | | | | | | | | | | | | | | | | |
